# Igniting Cold Tumors: Multi-Omics-Driven Strategies to Overcome Immune Evasion and Restore Immune Surveillance

**DOI:** 10.32604/or.2025.066805

**Published:** 2025-09-26

**Authors:** Xinyao Huang, Renjun Gu, Ziyun Li, Fangyu Wang

**Affiliations:** 1 The First Clinical Medical College, Nanjing University of Chinese Medicine, Nanjing, 210023, China; 2 School of Chinese Medicine, Nanjing University of Chinese Medicine, Nanjing, 210023, China; 3 Department of Gastroenterology and Hepatology, Jinling Hospital, Affiliated Hospital of Medical School, Nanjing University, Nanjing, 210016, China; 4 School of Acupuncture and Tuina, School of Regimen and Rehabilitation, Nanjing University of Chinese Medicine, Nanjing, 210023, China

**Keywords:** Cold tumors, multi-omics, immune evasion, tumor microenvironment, immune checkpoint inhibitors

## Abstract

Cold tumors, defined by insufficient immune cell infiltration and a highly immunosuppressive tumor microenvironment (TME), exhibit limited responsiveness to conventional immunotherapies. This review systematically summarizes the mechanisms of immune evasion and the therapeutic strategies for cold tumors as revealed by multi-omics technologies. By integrating genomic, transcriptomic, proteomic, metabolomic, and spatial multi-omics data, the review elucidates key immune evasion mechanisms, including activation of the WNT/β-catenin pathway, transforming growth factor-β (TGF-β)–mediated immunosuppression, metabolic reprogramming (e.g., lactate accumulation), and aberrant expression of immune checkpoint molecules. Furthermore, this review proposes multi-dimensional therapeutic strategies, such as targeting immunosuppressive pathways (e.g., programmed death-1 (PD-1)/programmed death-ligand 1 (PD-L1) inhibitors combined with TGF-β blockade), reshaping the TME through chemokine-based therapies, oncolytic viruses, and vascular normalization, and metabolic interventions (e.g., inhibition of lactate dehydrogenase A (LDHA) or glutaminase (GLS)). In addition, personalized neoantigen vaccines and engineered cell therapies (e.g., T cell receptor-engineered T (TCR-T) and natural killer (NK) cells) show promising potential. Emerging evidence also highlights the role of epigenetic regulation (e.g., histone deacetylase (HDAC) inhibitors) and N6-Methyladenosine (m6A) RNA modifications in reversing immune evasion. Despite the promising insights offered by multi-omics integration in guiding precision immunotherapy, challenges remain in clinical translation, including data heterogeneity, target-specific toxicity, and limitations in preclinical models. Future efforts should focus on coupling dynamic multi-omics technologies with intelligent therapeutic design to convert cold tumors into immunologically active (“hot”) microenvironments, ultimately facilitating breakthroughs in personalized immunotherapy.

## 1 Introduction

### 1.1 Immune Microenvironment of Cold Tumors

Cold tumors exhibit an immune-desert phenotype, characterized by a scarcity of tumor-infiltrating lymphocytes (TILs) and an accumulation of immunosuppressive cells, such as regulatory T cells (Tregs), which collectively impair antitumor immune responses [1]. Consequently, cold tumors are often difficult to treat and are associated with poor clinical outcomes. In contrast, hot tumors are defined by a high density of TILs, particularly CD8^+^ T cells, which mediate robust antitumor immune activity. This active immune surveillance helps inhibit tumor progression, leading to more favorable prognoses. The fundamental distinction between hot and cold tumors lies in the quantity and functional state of immune cells within the tumor microenvironment (TME) [2].

### 1.2 Core Mechanisms of Immune Evasion in Cold Tumors

**Figure 1:**
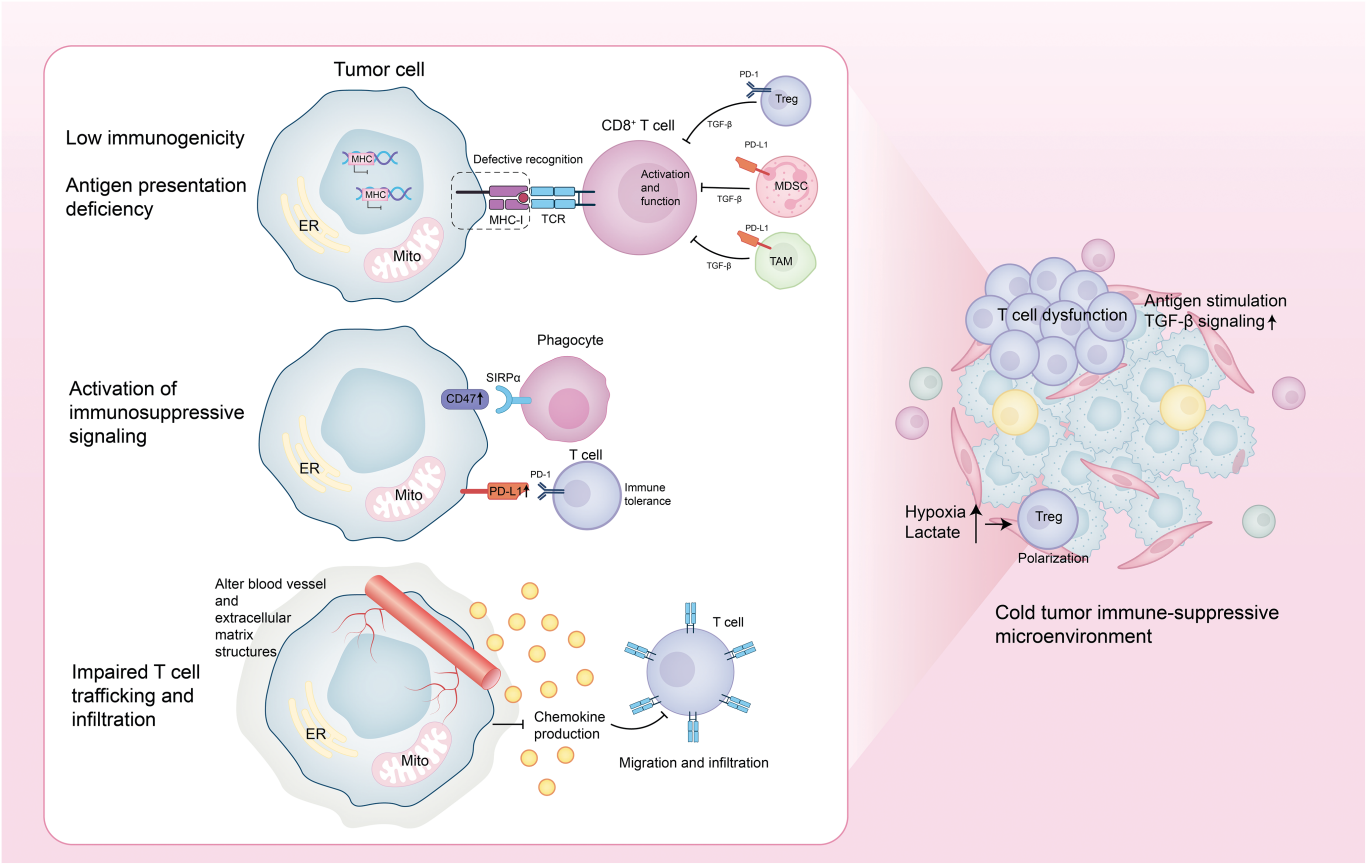
Core mechanisms of immune evasion in cold tumors. This figure was drawn using Adobe Illustrator 2024

Cold tumors evade immune surveillance and clearance through multiple interrelated mechanisms. First, they reduce antigen presentation by either lacking tumor-specific antigens or neoantigens and by downregulating or losing the expression of major histocompatibility complex class I (MHC-I) molecules, thereby impairing T cell recognition and activation. Second, cold tumors upregulate immune evasion molecules, such as the “don’t eat me” signal cluster of differentiation 47 (CD47) and the immunosuppressive ligand programmed death-ligand 1 (PD-L1), which inhibit both innate and adaptive immune responses. In addition, cold tumors suppress chemokine production, remodel the tumor vasculature and extracellular matrix (ECM), and physically restrict T cell migration and infiltration into the TME. Concurrently, immunosuppressive cells—including Tregs, myeloid-derived suppressor cells (MDSCs), and tumor-associated macrophages (TAMs)—accumulate within the TME. These cells secrete inhibitory cytokines (e.g., Transforming Growth Factor-beta (TGF-β)) and upregulate immune checkpoint molecules, further dampening T cell activation and function. Moreover, cold tumors induce T cell dysfunction through chronic antigen exposure and sustained TGF-β signaling, ultimately leading to T cell exhaustion, apoptosis, or ferroptosis. Metabolic alterations—such as lactate accumulation and tumor hypoxia—also contribute by impairing effector T cell activity and promoting the differentiation and expansion of immunosuppressive Tregs [1,3] (Fig. 1).

### 1.3 Necessity of Multi-Omics Technologies

Traditional single-dimensional approaches are insufficient to resolve the spatial and temporal complexity of the tumor immune microenvironment (TIME). Although single-cell technologies have revealed immune heterogeneity, they fall short in capturing cell–cell interactions and the spatial context. Computational deconvolution tools such as Estimating Relative Subsets Of RNA Transcripts (CIBERSORT) and Microenvironment Cell Populations-counter (MCP-counter) can estimate immune infiltration but fail to reflect the dynamic behavior of immune cells [4–6]. By contrast, the emergence of spatial multi-omics—integrating transcriptomic, proteomic, and metabolomic data—enables high-resolution mapping of tumor–immune crosstalk and identifies key transcriptional and metabolic alterations at the tumor–stroma interface. Single-cell profiling platforms, including Cytometry by Time-of-Flight (CyTOF), spatial transcriptomics, and multiplexed immunohistochemistry, further refine immune cell classification and spatial distribution within the TME [4–6]. Functional validation systems provide essential translational insight. For example, patient-derived organoids preserve the genomic, immune, and histological features of primary tumors, and when combined with single-cell sequencing, allow the comparison of immunological responses between responders and non-responders to immune checkpoint blockade (ICB). Additionally, co-culture models and humanized mouse systems have been used to verify mechanisms of immune suppression and therapeutic resistance [7]. In summary, multi-omics integration combined with functional platforms represents a powerful approach for decoding immune evasion mechanisms and advancing precision immunotherapy. These technologies are critical for resolving intratumoral heterogeneity, identifying targetable immune-suppressive pathways, and guiding the design of rational combination therapies.

This review provides a comprehensive analysis of immune evasion mechanisms in cold tumors and highlights multi-omics-driven strategies aimed at enhancing the efficacy of immunotherapy.

## 2 Multi-Omics Dissection of Immune Evasion

### 2.1 Genomics

Genomic technologies have significantly advanced our understanding of the immune evasion mechanisms underlying cold tumors, particularly in relation to tumor mutational burden (TMB) and the absence of neoantigens. Tumors with a high TMB typically harbor more neoantigens that can be recognized by the immune system, thereby triggering T cell activation and enhancing immune-mediated tumor elimination [8,9]. However, intratumoral heterogeneity (ITH) can result in the emergence of subclonal populations that escape immune surveillance, weakening the effectiveness of immunotherapy, especially when these subclones differ substantially in their antigenic profiles [9]. One key mechanism of immune evasion in cold tumors is the somatic loss of heterozygosity (LOH) at the HLA class I (HLA-I) locus. LOH impairs neoantigen presentation and enables immune escape, particularly in tumors with low TMB [10]. While these represent mechanisms of intrinsic tumor resistance, immune cell infiltration patterns reflect extrinsic microenvironmental suppression, which also independently contributes to immune evasion. Further analysis of colorectal cancer has identified an immune evasion-related gene signature (IEVSig) capable of predicting responses to immunotherapy. Patients with high IEVSig scores exhibit stronger immune evasion features and worse clinical outcomes [11]. Moreover, the epigenetic regulator EZH2 plays a dual role in immune escape: intrinsically, by suppressing genes essential for T cell activation; and extrinsically, by promoting the accumulation of immunosuppressive cells in the tumor microenvironment. Enhancer of Zeste Homolog 2 (EZH2) is closely associated with both TMB and immune checkpoint molecule expression, positioning it as a promising therapeutic target [12]. Collectively, these findings provide critical genomic insights into immune evasion in cold tumors and underscore the value of integrating TMB assessment, neoantigen profiling, and immune microenvironment characterization to improve the precision and efficacy of cancer immunotherapy [13].

More importantly, in-depth analyses based on genomic technologies have revealed that immune evasion in cold tumors is closely associated with key driver mutations, particularly the aberrant activation of the WNT/β-catenin signaling pathway. Data from large-scale genomic databases such as The Cancer Genome Atlas (TCGA) indicate that activation of this pathway suppresses chemokine expression and limits immune cell infiltration through intrinsic resistance mechanisms, thereby contributing to the formation of a “cold tumor” phenotype. Among 31 tumor types analyzed, approximately 90% of cases exhibited activation of the β-catenin pathway through gene mutations, somatic copy number alterations (SCNAs), or elevated protein expression levels [14]. This activation exerts consistent immunosuppressive effects across different cancers. For instance, in melanoma, it reduces T-cell infiltration by inhibiting the expression of chemokines such as Chemokine (C-C Motif) Ligand 4 (CCL4) and Chemokine (C-X-C Motif) Ligand 9/Chemokine (C-X-C Motif) Ligand 10 (CXCL9/CXCL10) [15]. In colorectal cancer, the pathway is significantly correlated with T-cell exclusion, independent of tumor mutational burden [16]. In hepatocellular carcinoma, mutations in exon 3 of β-catenin lead to downregulation of chemokines like Chemokine (C-C Motif) Ligand 20 (CCL20) and Chemokine (C-X-C Motif) Ligand 2 (CXCL2), thereby promoting an immunosuppressive microenvironment, a feature also observed in gliomas [17,18]. Beyond immunosuppression, WNT/β-catenin signaling exhibits bidirectional roles in tumor progression, highly dependent on tumor type, molecular subtype, and regulatory non-coding RNAs. In gastric cancer, for example, the AXIN1-295aa protein encoded by CircAXIN1 disrupts the β-catenin degradation complex and promotes tumor invasion, whereas circ-ITCH inhibits pathway activity by sponging miR-17 and upregulating ITCH expression [19,20]. In hepatocellular carcinoma, hsa_circ_104348 facilitates metastasis via the miR-187-3p/Rhotekin 2 (RTKN2) axis; circRNA-SORE induces resistance to sorafenib through miR-103a-2-5p/miR-660-3p; and hsa_circ_0004018 exerts tumor-promoting functions [21–23]. In prostate cancer, circABCC4 activates the pathway by stabilizing Cell Division Cycle and Apoptosis Regulator 1 (CCAR1) through N6-Methyladenosine (m6A) modification, while circPHF16 inhibits metastasis [24,25]. Mechanistically, endometrial cancer activates the pathway through either CTNNB1 exon 3 mutations, which prevent β-catenin degradation via phosphorylation, or Adenomatous Polyposis Coli protein (APC) inactivation, which disrupts the degradation complex [26]. In triple-negative breast cancer, downregulation of circ-ITCH lifts repression on miR-17 and miR-214, enhancing pathway activation, and in ovarian cancer, loss of circFBXO7 promotes β-catenin accumulation and Glycogen Synthase Kinase 3 Beta (GSK3β) phosphorylation [27,28]. Notably, therapeutic inhibition of the pathway—for example, by targeting Dickkopf-1 (DKK1) with DKN-01—can reshape the tumor immune microenvironment by restoring HLA/MHC expression [29]. Taken together, although the immunosuppressive consequences of WNT/β-catenin activation, such as reduced T-cell infiltration and impaired chemokine expression, represent extrinsic microenvironmental features, they are primarily driven by intrinsic tumor resistance mechanisms. Importantly, the pathway’s role in tumor progression is context-dependent and regulated by a complex network of molecular variants and non-coding RNAs. These findings underscore the paradoxical nature of the WNT/β-catenin pathway, which exhibits a consistent immunosuppressive signature across cancers, yet divergent roles in tumor progression depending on specific cellular and molecular contexts.

In addition, Clustered Regularly Interspaced Short Palindromic Repeats (CRISPR)/Cas9 technology has recently demonstrated immense potential in elucidating the immune evasion mechanisms of cold tumors. Through genome-wide CRISPR knockout screening, numerous key genes involved in tumor immune escape have been identified. For instance, in the context of cancer cell resistance to Chimeric Antigen Receptor T-Cell (CAR-T) cell-mediated cytotoxicity, autophagy-related genes such as ATG3, BECN1, and RB1CC1 were found to be enriched in tumor cells, facilitating immune evasion. Knockout of these genes significantly increased tumor cell susceptibility to CAR-T cells and improved therapeutic efficacy. Notably, elevated expression of these genes in tumor tissues was strongly associated with relapse and poor prognosis following Cluster of Differentiation 19 (CD19) CAR-T therapy [30]. In another study using the KPC3 pancreatic cancer model, immune evasion was found to be linked to epithelial-mesenchymal transition (EMT), with mesenchymal-like cancer cells exhibiting greater resistance to immune attack. CRISPR screening identified Epidermal Growth Factor Receptor **(**Egfr**)** and Milk Fat Globule-EGF Factor 8 **(**Mfge8**)** as critical mediators of this process [31]. Deletion of these genes enhanced the sensitivity of mesenchymal cancer cells to cytotoxic T lymphocytes (CTLs), and secreted Mfge8 was shown to inhibit CD8^+^ T cell proliferation and cytokine secretion, contributing to a more suppressive immune microenvironment [32]. Furthermore, CRISPR technology can be employed to disrupt immunosuppressive genes and upregulate MHC class I-related pathways, thereby enhancing tumor antigen presentation and improving immune cell recognition and cytotoxicity. When coupled with high-throughput CRISPR interference (CRISPRi) screening, this approach offers a powerful platform for uncovering novel regulators of immune evasion and identifying potential therapeutic targets [33]. Overall, these CRISPR-based studies primarily highlight mechanisms of intrinsic tumor resistance, although some targets, such as Mfge8, also play roles in extrinsic microenvironmental suppression. Collectively, these findings underscore the pivotal role of CRISPR technology in decoding the complex immune evasion landscape of cold tumors and provide a valuable resource for the development of next-generation immunotherapeutic strategies.

### 2.2 Transcriptomics

Through transcriptomic approaches, particularly single-cell RNA sequencing (scRNA-seq), the immune evasion mechanisms of cold tumors have been extensively elucidated, with a special focus on the TGF-β gene module associated with immunosuppression. In gastric cancer (GC) liver metastasis models, scRNA-seq revealed significant enrichment of immunosuppressive populations including cancer-associated fibroblasts (CAFs), MDSC-like macrophages, TAM-like macrophages, and naïve T cells, alongside marked reductions in conventional dendritic cells (cDCs) and effector CD8^+^ T cells [34]. TGF-β was identified as a key factor inhibiting NK cell activity, facilitating tumor immune escape and metastasis. Preclinical studies demonstrated that combining TGF-β inhibition with metastatic NK cell activation synergistically reduced liver metastases [34]. Further analyses showed TGF-β critically mediates interactions between CAFs and mesenchymal cancer cell subsets [35]. In multiple myeloma (MM), elevated TGF-β signaling within the tumor microenvironment correlated strongly with poor prognosis [36], while in lung adenocarcinoma (LUAD), high natural killer gene signature (NKGS) scores associated with TGF-β pathway genes predicted worse overall survival [37]. Targeting TGF-β signaling thus represents a promising therapeutic avenue for overcoming immune resistance and improving outcomes in cold tumor treatment.

Transcriptomic analyses have also highlighted the critical role of Vascular Endothelial Growth Factor (VEGF) in mediating immune evasion in both ovarian clear cell carcinoma (OCCC) and breast cancer (BC). In OCCC, VEGF promotes tumor progression primarily through angiogenesis and creation of an immunosuppressive microenvironment [38]. Elevated VEGF signaling in recurrent OCCC was closely associated with fibrosis and immune suppression, resulting in reduced T cell infiltration. High expression of Cluster of Differentiation 36 (CD36) and Cluster of Differentiation 47 (CD47) further intensified immune evasion [38]. Bevacizumab treatment not only remodeled tumor stroma but also restored T cell functionality [38]. Similarly, in breast cancer, the MCF-7 breast cancer cell secretome (MCF-7-CM) enhanced vascular endothelial cell proliferation while inhibiting lymphatic endothelial cell growth through VEGF-driven activation of ERK1/2 and Akt pathways [39]. Engineered T cells targeting mesothelin could transiently overcome VEGF-induced immunosuppression in ovarian cancer models [40].

Transcriptomic analyses have revealed critical defects in interferon (IFN) signaling pathways that contribute to immune evasion in cold tumors. Studies combining Perturb-Cellular Indexing of Transcriptomes and Epitopes by Sequencing (CITE) with CRISPR-Cas9 perturbation demonstrated that impaired interferon-γ (IFN-γ) signaling in melanoma-tumor-infiltrating lymphocyte (TIL) co-cultures compromises antigen presentation, facilitating immune escape [41]. Clinically, cold tumors exhibit reduced type I IFN signaling and defective antigen presentation, which correlates with low tumor mutational burden (TMB) and elevated somatic copy number variation (sCNV) scores [42].

The Cyclic GMP-AMP Synthase-Stimulator of Interferon Genes (cGAS-STING) pathway, a key mediator of IFN responses, is frequently suppressed in cold tumors. While this pathway normally detects cytoplasmic DNA to trigger IFN production and immune activation [43], key components (cGAS/STING) are often downregulated in cold tumors. The DNA translocase SWI/SNF Related, Matrix Associated, Actin Dependent Regulator of Chromatin, Subfamily a, Member 1 (SMARCAL1) has been identified as an intrinsic inhibitor of cGAS-STING activation, diminishing immune recognition of genomic instability [44]. Interestingly, in glioblastoma, OMA1-induced mitochondrial DNA release activates the pathway but ultimately suppresses T cell function through PD-L1 upregulation [45]. Additional tumor-specific regulatory mechanisms include PCBP2-mediated pathway suppression in prostate cancer [46] and IFNλ receptor deficiency in non-small cell lung cancer [47]. Dysregulation of interferon-stimulated genes (ISGs) further exacerbates immune evasion through multiple molecular mechanisms. The polycomb repressive complex component CBX2 recruits HDAC10 to suppress H3K27ac modification at ISG promoters [48], while SOX2 maintains ISG resistance by repressing SOCS3 and PTPN1 transcription [49]. In breast cancer, JMJD8 inhibits type I IFN signaling by blocking STING-TBK1 complex formation [50], and progesterone receptor (PR) interacts with STAT1 to suppress IFN-induced STAT1 phosphorylation [51]. The long non-coding RNA VPS9D1-AS1 in colorectal cancer enhances TGF-β/ISG signaling to inhibit CD8^+^ T cell infiltration [52], whereas SMARCA4 deletion in ovarian cancer upregulates ISGs to enhance antigen presentation and tumor immunogenicity [53].These findings collectively demonstrate how diverse defects in interferon signaling pathways create an immunosuppressive tumor microenvironment that facilitates immune evasion across multiple cold tumor types, providing a strong rationale for developing therapies targeting these pathways.

Transcriptomic studies have elucidated the critical role of CD8^+^ T cell exhaustion in promoting immune evasion in cold tumors. In bladder cancer, single-cell analyses revealed a progressive differentiation trajectory from TCF7^+^ precursor cells to terminally exhausted STMN1^+^ CD8^+^ T cells, characterized by gradual loss of effector functions [54]. Chronic antigen exposure drives this exhaustion through NFAT- and NR4A-mediated transcriptional reprogramming [55–57], with additional contributions from Polybromo-associated BRG1-associated factor complex (PBAF), Nuclear Factor kappa-light-chain-enhancer of activated B cells (NF-κB), Nuclear Factor of Activated T-cells, Cytoplasmic 2 (NFATC2), Nuclear Receptor Subfamily 4 Group A Member 1 (NR4A1), One Cut Homeobox 2 (ONECUT2), and Ets Variant 4 (ETV4), along with signaling pathways such as Interleukin 18 Receptor (IL18R) and C-X-C Motif Chemokine Receptor 4 (CXCR4) transcription factors that upregulate inhibitory receptors while suppressing cytotoxic molecules [58–61]. Notably, while precursor exhausted T cells remain responsive to checkpoint blockade, terminally exhausted populations show limited therapeutic sensitivity [62]. Ectopic TCF1 expression has been shown to prevent terminal exhaustion by modulating B lymphocyte-induced maturation protein 1/Bcl-2 interacting mediator of cell death (Blimp1/Bim) and Tox Family Transcription Factor 2/Runt-Related Transcription Factor 3 pathways [63]. Parallel transcriptomic analyses have identified myeloid cell heterogeneity as another key contributor to immunosuppression. In bladder cancer, tumor-associated macrophages (TAMs) adopt a pro-tumor phenotype while dendritic cells differentiate into immunosuppressive LAMP3^+^ subsets [54]. In lung squamous cell carcinoma, TAM-derived migration inhibitory factor (MIF) activates the Phosphoinositide 3-Kinase (PI3K)-STAT3-PD-L1 axis to suppress CD8^+^ T cell activity [64]. Similar mechanisms occur in plasmacytoid dendritic cell tumors, where T cell exhaustion accompanies clonal expansion [65]. Gallbladder cancers show increased TAM infiltration associated with T cell dysfunction [66], while follicular helper T cell lymphomas exhibit clonal evolution-driven crosstalk between tumor cells, exhausted T cells, and myeloid populations [67]. Clear cell renal carcinoma demonstrates synergistic immune evasion through interactions between diverse myeloid subsets and tumor cell copy number variations [68]. Overall, transcriptomic studies have highlighted the pivotal roles of CD8^+^ T-cell exhaustion trajectories and myeloid cell heterogeneity in driving immune evasion across multiple cold tumor types, providing a theoretical foundation and potential strategies for targeted remodeling of immune cell states.

In addition to T cell dysfunction, the failure of antigen-presenting cell (APC) activation, particularly dendritic cells (DCs), also represents a critical mechanism of immune evasion in cold tumors. The absence of cross-presenting competent Conventional Dendritic Cell 1 (cDC1) subsets (e.g., CD103^+^ and CD11b^+^ DCs) in cold tumors impairs the effective priming of naïve T cells [69]. In osteosarcoma, mature regulatory DCs (mregDCs), which originate from cDC1s, upregulate co-inhibitory molecules and secrete chemokines that recruit Tregs, establishing an immunosuppressive microenvironment and further inhibiting DC activation [70]. Moreover, tumor-derived immunosuppressive factors actively inhibit DC differentiation and function. In melanoma, the tumor microenvironment induces the upregulation of Interleukin 6 Receptor (IL-6R) and Interleukin 10 Receptor (IL-10R) on DCs, activating the STAT3 signaling pathway and suppressing the Toll-like receptor (TLR)-driven maturation program. This results in impaired DC priming capacity, defective migration, and insufficient antigen presentation [71]. Tumor factors can also activate the transcription factor Activating Transcription Factor 3 (ATF3), which suppresses the expression of cholesterol 25-hydroxylase (CH25H), thereby disrupting antigen processing and weakening cross-presentation [72]. In cervical squamous cell carcinoma, DCs express immunosuppressive molecules such as indoleamine 2,3-dioxygenase 1 (IDO1) and galectin-9 (LGALS9), while exhibiting low PD-L1 expression, revealing spatially heterogeneous mechanisms of immune suppression [73]. Additionally, DCs are often sequestered at the tumor periphery, limiting their interaction with intratumoral T cells. For example, in intrahepatic cholangiocarcinoma, cDC1s predominantly accumulate in non-tumorous regions and are markedly reduced near the tumor, leading to a naïve and functionally inert phenotype in CD8^+^ T cells [74]. Collectively, these initiation defects significantly hinder effective T cell activation and represent a major obstacle to successful immunotherapy in cold tumors.

### 2.3 Proteomics

Proteomic studies have revealed the pivotal role of immune checkpoint proteins in facilitating immune evasion in cold tumors. In bladder cancer, loss of the Y chromosome (LOY) has been shown to intrinsically impair CD8^+^ T cell function, leading to dysfunction and exhaustion, thereby promoting immune evasion. Interestingly, LOY mutations also enhance tumor responsiveness to programmed death-1 (PD-1) immune checkpoint blockade therapy, suggesting that such mutations may sensitize tumors to immunotherapy. This finding offers novel insights into the identification of immunotherapy targets and predictive biomarkers [75]. In natural killer/T-cell lymphoma (NKTCL), the protein S100 Calcium-Binding Protein A9 promotes intrinsic immune evasion by upregulating PD-L1 expression through activation of the ERK1/2 signaling pathway. Simultaneously, S100A9 contributes to extrinsic immune suppression by inducing the accumulation of myeloid-derived suppressor cells (MDSCs). Pharmacological inhibition of the ERK1/2 pathway significantly reduced S100A9-mediated immune evasion and tumor progression [76].

Recent studies have also highlighted the critical role of intercellular communication in driving immune evasion in cold tumors. Proteomic analyses have revealed that the extracellular matrix (ECM), as a central hub of soluble and structural signals, orchestrates bidirectional interactions between tumor cells and immune cells, particularly T lymphocytes. Mass spectrometry–based characterization of ECM components has identified key proteins and remodeling enzymes that regulate T cell infiltration, cytokine secretion, and immune suppression [77]. Moreover, exosomes have been shown to mediate intrinsic immune evasion by modulating tumor cell metabolism. They transport glycolytic enzymes and metabolites that reprogram tumor metabolic pathways, enhancing glucose uptake and lactate production. This metabolic shift supports tumor cell survival and proliferation, while simultaneously contributing to an immunosuppressive microenvironment that facilitates immune evasion [78].

Moreover, proteomic studies have unveiled the critical role of kinase activity regulation in mediating immune evasion in cold tumors, particularly in Human Papillomavirus-associated malignancies. The ErbB2 receptor tyrosine kinase (HER2) has been shown to regulate the activity of the HPV16 long control region (LCR), thereby promoting the activation of the viral promoter and enhancing the expression of the HPV oncogenes E6 and E7 [79]. Through the Akt and ERK signaling pathways, ErbB2 kinase activity intrinsically facilitates immune evasion by upregulating viral oncogene expression [79]. In mucinous adenocarcinoma (MC), proteomic analyses identified 846 differentially expressed proteins in serum-derived extracellular vesicles from MC patients. These proteins were strongly associated with cellular migration, tumor microenvironment remodeling, and immune evasion pathways. Notably, PLA2G2A was significantly upregulated in MC and was found to promote the invasion and migration of SW480 colon cancer cells by activating the Wnt/β-Catenin signaling pathway, thus driving MC malignancy. High PLA2G2A expression was also associated with poor prognosis in colon cancer patients harboring BRAF mutations, suggesting its potential as both a prognostic biomarker and a novel immunotherapy target [80]. Furthermore, the regulation of kinase activity through the laminin receptor precursor/laminin receptor (LRP/LR) plays a key role in immune evasion in lung cancer. Downregulation of LRP/LR in A549 lung cancer cells significantly reduced cell viability, migratory capacity, and telomerase activity, while promoting apoptosis. Proteomic analyses indicated that silencing LRP/LR restored the intrinsic sensitivity of tumor cells to immune responses, further emphasizing its role in promoting immune evasion [81].

### 2.4 Metabolomics

Recent studies have extensively investigated the relationship between metabolic reprogramming and immune evasion across various tumor types. Utilizing liquid chromatography-mass spectrometry (LC-MS/Q-TOF and LC-MS/QQQ), significant alterations in metabolites such as tryptophan, methionine, and kynurenine were identified in glioblastoma (GBM) cells and tissues compared to normal human astrocytes (NHA). Notably, GBM cells exhibit a strong dependence on dietary methionine to sustain proliferation, colony formation, and a modified methylation profile, reflected by an altered S-adenosylmethionine/S-adenosylhomocysteine (SAM/SAH) ratio [82]. Furthermore, the aberrant activation of the kynurenine pathway intrinsically contributes to immune evasion by impairing T cell function, thereby enhancing the tumor’s resistance to immune surveillance [82]. In hepatocellular carcinoma, intrinsic loss of the histone variant macroH2A1 promotes the proliferation of cancer stem-like cells and facilitates immune evasion by modulating downstream immunosuppressive signaling pathways. These cells also exert extrinsic immunosuppressive effects by activating Tregs, thereby suppressing effector T cell responses and dampening anti-tumor immunity [83]. In addition, *de novo* sphingolipid biosynthesis has emerged as a critical intrinsic metabolic adaptation supporting tumor progression and immune escape. Lipidomic profiling revealed that inhibition of sphingolipid synthesis enhances the cytotoxic activity of NK cells and CD8^+^ T cells, partly through activation of the IFN-γ signaling pathway, thereby restoring anti-tumor immunity [84]. Lastly, metabolomic analyses demonstrated that intrinsic metabolic rewiring, such as upregulation of adenosine deaminase and phosphoinositide-dependent kinase-1 (PDK1), can improve CAR-T cell infiltration and antitumor efficacy, ultimately overcoming extrinsic immunosuppressive barriers in cold tumors [85].

### 2.5 Temporal Dynamics

Temporal kinetic analyses have revealed that tumor immune escape is a dynamic and evolving process, primarily driven by therapy-induced immunoediting and adaptive resistance. The central mechanism involves the progressive loss of immunogenic mutations or high-quality neoantigens due to therapeutic pressure or epigenetic silencing. Concurrently, tumors suppress antigen presentation by inhibiting IFN-γ signaling and activating immunosuppressive pathways, thereby establishing a selective immune escape advantage [86]. Among these regulatory factors, YTH N6-methyladenosine RNA binding protein 1 (YTHDF1) plays a pivotal role in immunoediting. Its expression increases with tumor progression and is significantly correlated with reduced CD8^+^ T cell infiltration, the emergence of an “immune desert” phenotype, and resistance to immune checkpoint inhibitors (ICIs). Conversely, YTHDF1 deficiency activates CD8^+^ T cell cytotoxicity pathways and suppresses tumorigenesis, underscoring its role in maintaining an immunosuppressive tumor microenvironment through dynamic immune editing [87]. The Tumor Evasion and Antigen Loss model further quantifies this process, demonstrating that a pro-tumor microenvironment accelerates immune escape through early immunoevasion or depletion of tumor-associated antigens, whereas an anti-tumor microenvironment tends to accumulate more recognizable antigens prior to immune escape, thereby enhancing immune plasticity [88]. The temporal and spatial heterogeneity of TAMs reflects the impact of immune editing over time, with a phenotypic shift from M1 to M2 macrophages facilitating tumor immune escape [89]. Temporal proteomics using the Mouse Forestomach Carcinoma model revealed a three-phase immune dynamic: an early-stage activation of innate and adaptive immunity (day 3), a phase of robust immune surveillance (days 7–14) marked by MHC-I/II upregulation and cytotoxic T/NK cell activation, and a late-stage immune escape (day 21) characterized by extracellular matrix remodeling and dysregulation of antigen processing mechanisms [90]. Epigenetic regulation further drives this temporal evolution. Tumors with high alternative promoter load exhibit time-dependent resistance to immunotherapy and shortened progression-free survival. This is mediated by dynamic modulation of the immune microenvironment, leading to functional decline of T cells [91]. Collectively, these findings highlight the multidimensional nature of adaptive resistance and reveal how therapy-induced immunoediting orchestrates the temporal dynamics of tumor immune escape.

### 2.6 Spatial Multi-Omics

Spatial transcriptomics enables high-resolution mapping of immune cell–tumor cell interactions, thereby elucidating the mechanisms of immune evasion and informing the optimization of immunotherapeutic strategies. For instance, in hepatocellular carcinoma (HCC), intrinsic upregulation of high mobility group box 2 (HMGB2) is associated with T cell exhaustion within an extrinsically immunosuppressive microenvironment [92]. Similarly, in esophageal squamous cell carcinoma (ESCC), intrinsic tumor subclonal evolution is shaped by extrinsic immune selection pressure, underscoring their synergistic role in driving immune escape [93]. In GC, intrinsic overexpression of C-X-C motif chemokine receptor 4 (CXCR4) is linked to Treg exhaustion and promotes immune evasion through oxidative stress and NF-κB activation [94]. Additionally, intrinsic expression of CCL5, Chemokine (C-C Motif) Ligand 5 (CCL5) in circulating tumor cells recruits Tregs via activation of the TGF-β1-p38-MAX signaling pathway, thereby facilitating metastasis and immune escape [95]. Integrated multi-omics approaches further clarify how the metabolic microenvironment modulates the spatial distribution and function of immune cells. In HCC, malignant cells alter tryptophan metabolism to impair the maturation of tertiary lymphoid structures (TLS), thus reducing the efficacy of immune checkpoint blockade (ICB); metabolic intervention can restore TLS development and enhance antitumor immunity [96]. In ESCC, creatine accumulation combined with loss of hexokinase 3 (HK3) synergistically promotes M2 polarization of tumor-associated macrophages, contributing to the establishment of an immunosuppressive microenvironment [97]. In stage I non-small cell lung cancer (NSCLC), genomic instability and DNA hypomethylation lead to upregulation of the cancer-testis antigen Preferentially Expressed Antigen in Melanoma, disrupting interactions between CD8^+^ T cells and alveolar type II epithelial cells, and fostering an immunosuppressive niche that predisposes to postoperative recurrence [98]. Spatial metabolomics, lipidomics, and transcriptomics in GC reveal pronounced molecular heterogeneity: genes and metabolites related to fatty acid synthesis are enriched in tumor cores, whereas immune-regulatory lipids and associated genes are elevated in lymphoid regions, suggesting spatial lipid metabolic reprogramming supports both tumor cell viability and immune modulation [5]. In oral squamous cell carcinoma (OSCC), metabolic reprogramming drives fibroblast transformation into inflammatory cancer-associated fibroblasts (iCAFs) via Hypoxia-Inducible Factor 1 Alpha (HIF1A)-mediated CXCL12 expression. These iCAFs recruit Tregs, forming an epithelial cell–iCAF–Treg axis that reflects the metabolic-immune synergy in remodeling the TME [99]. Collectively, spatial multi-omics analyses illuminate how intrinsic genomic and metabolic reprogramming fosters immune evasion, while extrinsic immunosuppressive microenvironments promote tumor progression through coordinated metabolic and intercellular signaling networks. These insights provide a refined framework for identifying precise immunotherapeutic targets in cold tumors.

**Figure 2:**
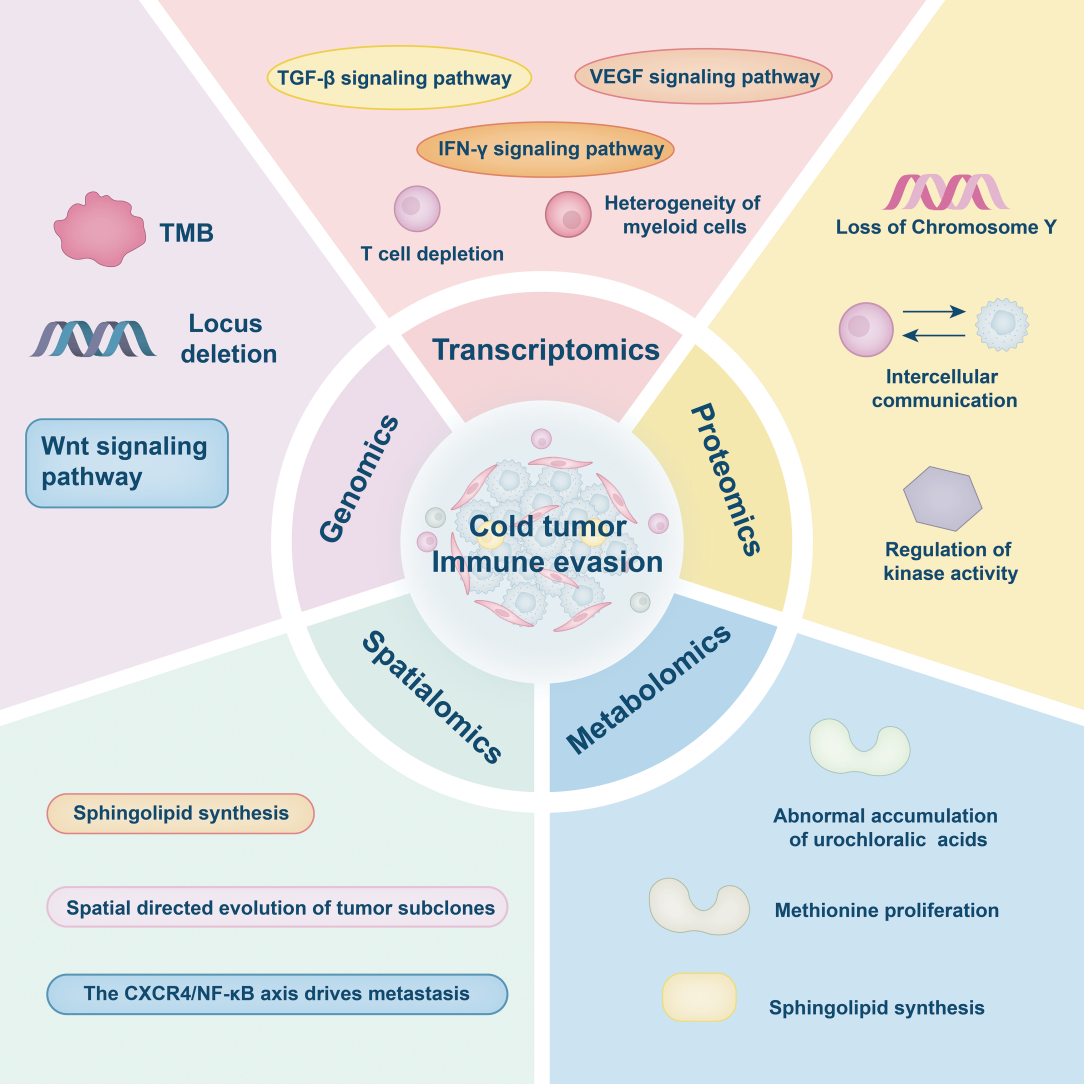
Multi-omics dissection of immune evasion. This figure was drawn using Adobe Illustrator 2024

Despite its capacity to depict the heterogeneity of the TME, spatial transcriptomics lacks the resolution necessary to determine functional suppression between specific cell types. For instance, in primary colorectal cancer, although the technique can identify the spatial co-localization of CD4^+^ and CD8^+^ T cells alongside the upregulation of immune checkpoint genes, it cannot directly demonstrate whether CD4^+^ T cells functionally suppress CD8^+^ T cell activity, nor can it accurately resolve the spatial and temporal evolution of immunosuppressive processes [100]. Similarly, in NSCLC, while spatial transcriptomics can delineate the distribution patterns of TAMs and correlate them with clinical prognosis, heterogeneity in the expression of genes such as Colony Stimulating Factor 1 Receptor (CSF1R) indicates functional diversity within TAM subsets. However, the specific roles of these subpopulations remain elusive due to the limitations of current spatial technologies [101,102]. Furthermore, transcriptomic data alone are insufficient to differentiate whether observed metabolic alterations arise from tumor-intrinsic regulatory mechanisms or from extrinsic microenvironmental influences, thereby constraining the ability to infer mechanistic causality [96]. Although spatial multi-omics technologies have significantly advanced the depth and breadth of TME research by integrating transcriptomic, proteomic, and metabolomic data, they still face substantial limitations related to data quality, spatial resolution, and clinical applicability. At the mechanistic level, integration of proteomic and metabolomic data remains constrained by low detection sensitivity and poor stability of metabolites, resulting in incomplete characterization of critical immune-metabolic regulatory networks [97]. From a technological application standpoint, issues such as small sample sizes, limited resolution for distinguishing cellular subtypes (e.g., specific CD4^+^ T cell subsets), and a lack of matched samples from relapsed or treatment-resistant tumors weaken the clinical relevance and generalizability of current findings [98,99]. Moreover, the inherent heterogeneity and complexity of multi-omics data present significant challenges for cross-platform integration. There is a pressing need to establish standardized analytical workflows and quality control measures to enhance data reproducibility, improve inter-study comparability, and ultimately increase the translational value of spatial multi-omics research in oncology (Fig. 2) (Table 1).

**Table 1:** Multi-omics dissection of immune evasion

Omics technology	Core findings	Molecular mechanism/pathway	Functional impact	Relevant cancer types	Clinical significance	References
Genomics	High TMB correlates with neoantigen load. ITH and HLA-I LOH promote immune escape	WNT/β-catenin pathway activation; Autophagy-related genes	Intrinsic resistance via neoantigen loss; Extrinsic suppression via immune exclusion	Melanoma, CRC, HCC, Glioma	Prognostic IEVSig model; CRISPR-edited targets improve CAR-T efficacy	[8,10,14,18,23,30]
Transcriptomics	TGF-β/VEGF modules drive immunosuppression; IFN/cGAS-STING defects impair immunity	TGF-β-NK cell inhibition; VEGF-angiogenesis	CAF/TAM-mediated T-cell exclusion; ISG downregulation	OCCC, BC, LUAD, MM	Bevacizumab restores T-cell function. IFN-γ pathway targeting reverses immune evasion	[34,37,41,44,49]
Proteomics	Immune checkpoint (PD-L1) upregulation; Kinase (HER2, LRP/LR) activity	ERK1/2-PD-L1 axis; HPV oncogene regulation	CD8^+^ T-cell exhaustion; Viral immune evasion	Bladder cancer, NKTCL, HPV^+^ cancers	LOY mutations predict PD-1 response; PLA2G2A as a BRAF-mutant CRC biomarker	[75,76,78]
Metabolomics	Tryptophan /kynurenine and sphingolipid dysregulation	Kynurenine-T cell suppression; SAM/SAH imbalance	Metabolic competition with T cells; Stemness maintenance	GBM, HCC	Methionine restriction sensitizes to immunotherapy	[82–85]
Temporal dynamics	Immunoediting depletes neoantigens; YTHDF1 mediates adaptive resistance	IFN-γ signaling loss; Epigenetic silencing	Dynamic immune desert formation	Pan-cancer	YTHDF1 deficiency enhances ICB response	[86,87,90]
Spatial multi- omics	HMGB2/CXCR4-CCL5 axis recruits Tregs; Metabolic TLS impairment	p38-MAX-Treg recruitment; HK3-creatine-M2 polarization	Spatial immune exclusion; Altered antigen presentation	HCC, ESCC, GC, NSCLC	Metabolic modulation restores TLS function; PRAME-targeted therapy prevents recurrence	[92,95,97]

Note: TMB, Tumor mutational burden; ITH, Intratumoral heterogeneity; HLA-I, Human Leukocyte Antigen Class I; LOH, Loss of heterozygosity; WNT, Wingless; CRC, Colorectal Cancer; HCC, Hepatocellular carcinoma; IEVSig, Immune evasion signature; CAR-T, Chimeric Antigen Receptor T-Cell; TGF-β, Transforming Growth Factor-beta; VEGF, Vascular Endothelial Growth Factor; IFN, Interferon; cGAS-STING, Cyclic GMP-AMP Synthase-Stimulator of Interferon Genes; NK, Natural Killer cells; CAF, Cancer-associated fibroblasts; TAM, Tumor-associated macrophage; ISG, Interferon-stimulated genes; OCCC, Ovarian clear cell carcinoma; BC, Breast cancer; LUAD, Lung adenocarcinoma; MM, Multiple myeloma; IFN-γ, Interferon-γ; PD-L1, Programmed cell death ligand 1; HER2, The ErbB2 receptor tyrosine kinase; LRP/LR, Laminin receptor precursor/laminin receptor; ERK1/2, Extracellular Signal-Regulated Kinase 1/2; NKTCL, NK/T cell lymphoma; LOY, Y chromosome loss; SAM/SAH, S-adenosylmethionine/S-adenosylhomocysteine; GBM, Glioblastoma; YTHDF1, YTH N6-methyladenosine RNA binding protein 1; HMGB2, High mobility group box 2; CXCR4, C-X-C motif chemokine receptor 4; CCL5, Chemokine (C-C Motif) Ligand 5; ESCC, Esophageal squamous cell carcinoma; GC, Gastric Cancer; NSCLC, Non-small cell lung cancer; TLS, Tertiary lymphoid structures; PRAME, Preferentially Expressed Antigen in Melanoma.

## 3 Therapeutic Intervention Strategies

**Figure 3:**
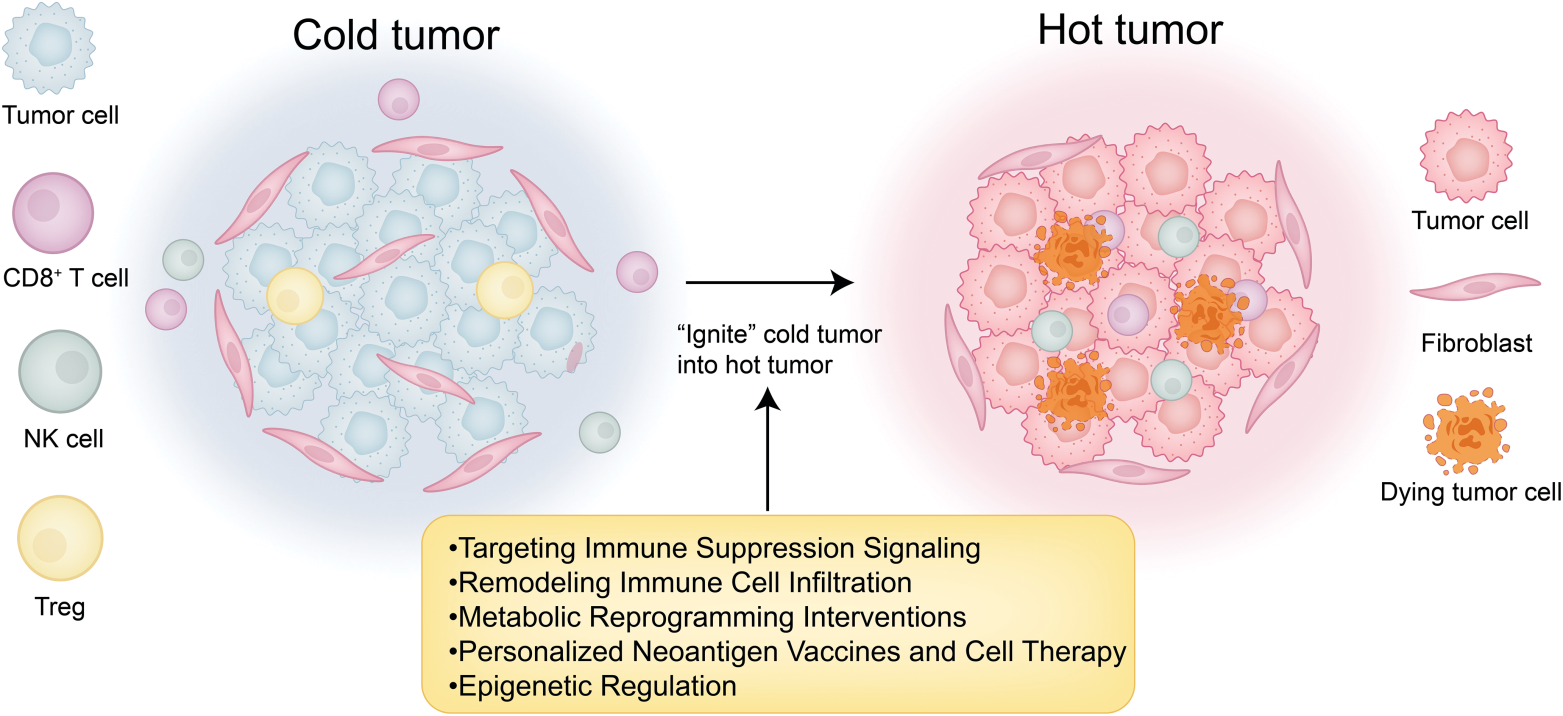
“Cold-to-hot” transition map. This figure was drawn using Adobe Illustrator 2024

To convert cold tumors into hot tumors, key strategies include targeting immune suppression signaling, remodeling immune cell infiltration, metabolic reprogramming to improve immune function, deploying personalized neoantigen vaccines and cell therapies, and applying epigenetic regulation to modulate immune responses. These interventions aim to transform immunologically inert tumors into inflamed, therapy-responsive hot tumors (Fig. 3).

### 3.1 Targeting Immune Suppression Signaling

Cold tumors exhibit limited responsiveness to immunotherapy, primarily due to their immunosuppressive TME. Consequently, targeting key immunosuppressive signaling pathways has emerged as a critical therapeutic strategy. The combination of ICIs with other targeted therapies has shown promise in enhancing antitumor immune responses by disrupting extrinsic immune evasion mechanisms mediated by the TME. In addition, intrinsic metabolic pathways within tumor cells, such as ferroptosis and tryptophan metabolism, play pivotal roles in shaping the immune microenvironment. Therapeutically targeting these metabolic pathways can effectively modulate immune responses and improve the efficacy of immunotherapy. Moreover, the adenosine signaling pathway, an important extrinsic contributor to immune suppression in the TME, plays a vital role in tumor immune escape. Inhibition of adenosine production and blockade of its receptors can relieve immunosuppression and further augment the therapeutic benefits of immunotherapy.

#### 3.1.1 Combination with Immune Checkpoint Inhibitors

To better understand intervention strategies for cold tumor treatment, this section focuses on the application of ICIs. Cold tumors are characterized by an immunosuppressive tumor microenvironment, which limits the effectiveness of immunotherapeutic approaches. Therefore, identifying strategies to “warm up” cold tumors is essential for enhancing treatment outcomes. Several studies have highlighted potential targets for overcoming intrinsic and extrinsic immune resistance mechanisms. For instance, aberrant expression of glutamate decarboxylase 1 (GAD1) in tumor cells has been shown to mediate intrinsic resistance by promoting tumor cell proliferation via β-catenin activation. Concurrently, GAD1 contributes to extrinsic immune suppression by reducing CD8^+^ T cell infiltration. Targeting GAD1 or the associated Gamma-Aminobutyric Acid (GABA) signaling pathway can enhance T cell infiltration into tumors, thereby improving the efficacy of immunotherapy [103]. Additionally, DKK1 regulates the immunosuppressive phenotype of TAMs, representing an extrinsic mechanism that impairs CD8^+^ T cell-mediated antitumor activity. Inhibiting DKK1 can reprogram TAMs, boost immune responses, and enhance the therapeutic effectiveness of PD-1 blockade [104]. In HCC, CTNNB1 mutations intrinsically upregulate matrix metalloproteinase 9 (MMP9), which in turn mediates extrinsic suppression by hindering CD8^+^ T cell infiltration, contributing to immune evasion. Targeting MMP9 restores T cell functionality and improves the response to anti-PD-1 therapy [105]. Moreover, CAFs have been found to secrete WNT2, which inhibits DC differentiation. Targeting WNT2 can restore DC functionality and enhance immune responses [106]. Low-density lipoprotein receptor-related protein 1 (LRP1) promotes M2 macrophage polarization via activation of the NOTCH signaling pathway; inhibiting LRP1 can re-establish antitumor immune responses and help overcome resistance to ICIs [107]. In prostate cancer, Yes-associated protein 1 (YAP1) has been implicated in maintaining CAF phenotypes that support immune evasion. Targeting YAP1 can reprogram CAFs, thereby enhancing immune infiltration and improving immunotherapeutic efficacy [108]. In addition, poly(A) binding protein cytoplasmic 1-like (PABPC1L) promotes T cell dysfunction and Treg infiltration by upregulating IDO1; targeting PABPC1L may improve antitumor immunity and enhance anti-PD-1 treatment responses [109]. Finally, FtsJ RNA 2^′^-O-methyltransferase 3 (FTSJ3) expression is upregulated in HCC, where it suppresses antitumor immune responses. Inhibition of FTSJ3 activates the type I interferon pathway and potentiates immunotherapy efficacy [110].

Recent studies have identified increasingly precise strategies targeting immune evasion mechanisms within the tumor immune microenvironment. The PD-1/PD-L1 signaling pathway plays a pivotal immunosuppressive role in the tumor microenvironment, with exosomal PD-L1 (exoPD-L1) facilitating immune escape by inhibiting antitumor immune responses. Targeting exoPD-L1 has been demonstrated to enhance immunotherapy efficacy [111]. Moreover, patients harboring loss-of-function mutations in Neurotrophic Receptor Tyrosine Kinase 1 (NTRK1) exhibit improved responses to immunotherapy, and inhibition of the NTRK1 signaling pathway significantly potentiates the effectiveness of immune checkpoint inhibitors [112]. In HCC, the combination of anlotinib and anti-PD-1 therapy targets the transferrin receptor, thereby overcoming immune resistance and improving therapeutic outcomes [113]. Echinacoside has been shown to downregulate PD-L1 expression and promote T cell infiltration; when combined with ICIs, it markedly enhances immunotherapy efficacy [114]. Liposomal formulations of perilla alcohol improve its solubility and bioavailability, demonstrating pronounced antitumor effects in combination with anti-PD-1 therapy, effectively reversing immune resistance [115]. Furthermore, inhibition of PTPN3 activates T cell function and augments the therapeutic efficacy of anti-PD-1 treatment in cold tumors under hypoxic conditions [116]. Studies have also revealed that anti-PD-1 therapy modulates Treg function in melanoma, alleviating suppression on CD8^+^ T cells and thereby improving immunotherapeutic outcomes [117]. In summary, targeting immune-suppressive signaling pathways, particularly those mediating extrinsic mechanisms, combined with immune checkpoint inhibition, offers a promising intervention strategy to overcome resistance and enhance the efficacy of immunotherapy in cold tumors.

**Figure 4:**
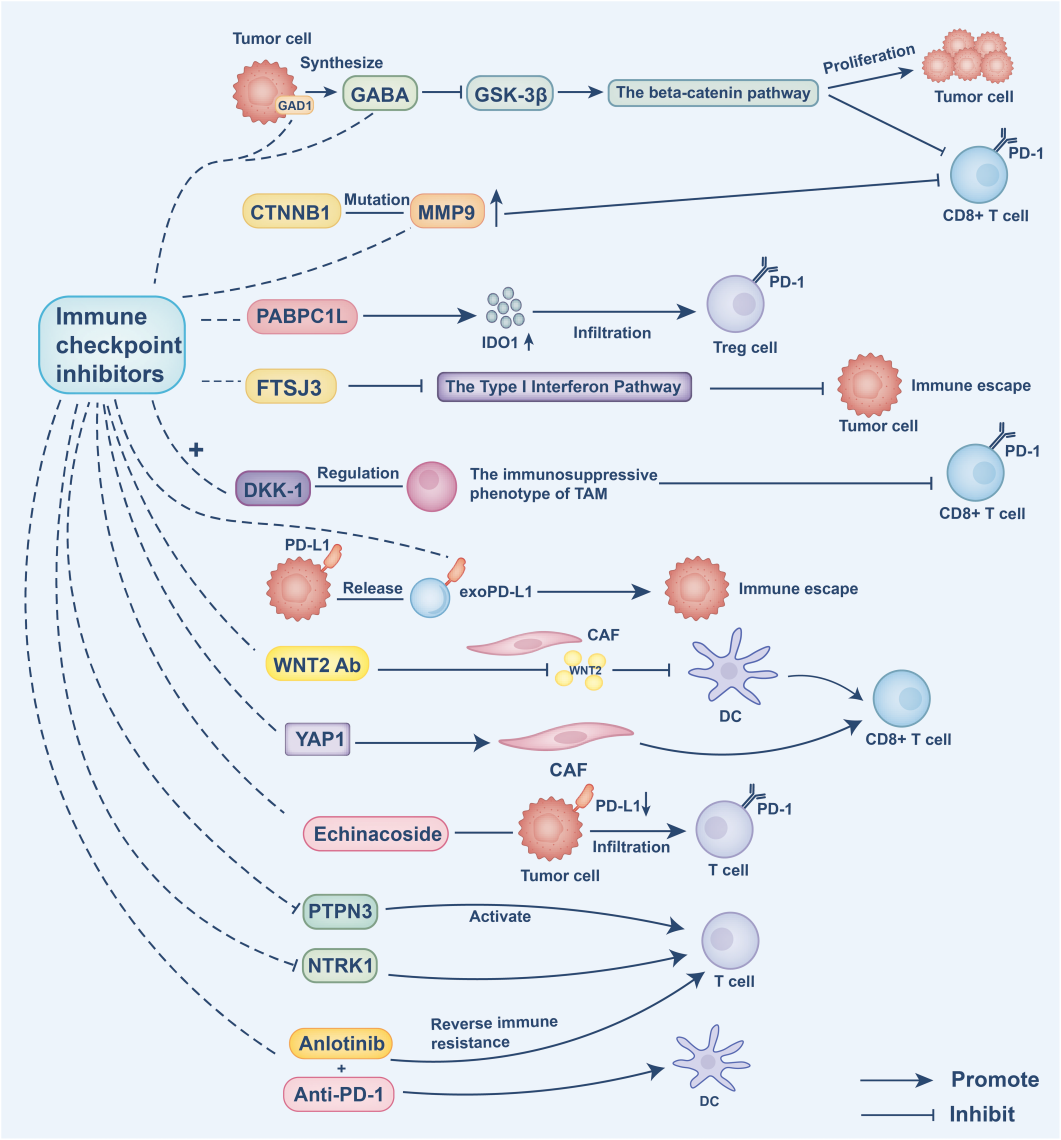
Intrinsic resistance mechanisms: Tumor cells aberrantly express GAD1, promoting GABA synthesis, which inhibits GSK-3β and activates β-catenin signaling. This drives tumor cell proliferation and reduces CD8^+^ T cell antitumor activity. CTNNB1 mutations intrinsically upregulate matrix metalloproteinase 9 (MMP9), which in turn mediates extrinsic suppression by hindering CD8^+^ T cell infiltration, contributing to immune evasion. PABPC1L intrinsically enhances IDO1 expression, leading to T cell dysfunction and regulatory T cell (Treg) infiltration. FTSJ3 expression inhibits type I interferon responses, impairing T cell activity. Inhibition of these intrinsic pathways restores T cell function and enhances the efficacy of ICIs

Current ICIs combined with other targeted therapies still face significant limitations. For example, DKK1 blockade encounters challenges related to targeting precision and therapeutic resistance, due to the complex origin of DKK1, its reliance on PI3K-AKT signaling, and the existence of immunocompensatory mechanisms [104]. Although combining anti-WNT2 with anti-PD-1 therapy shows promise, targeting cancer-associated fibroblasts (CAFs) may disrupt the tumor barrier, and the tissue-specific regulation of the WNT2 pathway increases the risk of adverse effects during clinical translation [106]. Moreover, the heterogeneity and plasticity of CAFs present a therapeutic opportunity, yet insufficient identification of CAF subtypes and the lack of specific markers for key regulators such as YAP1 constrain its clinical application [108]. Additionally, the efficacy of anlotinib combined with PD-1 blockade remains limited to preclinical studies, with potential risks of combination toxicity and off-target effects yet to be fully evaluated [113] (Fig. 4).

Extrinsic immune suppression mechanisms: DKK1 shapes the immunosuppressive phenotype of tumor-associated macrophages (TAMs), limiting CD8^+^ T cell infiltration. Tumor-derived exosomal PD-L1 (exoPD-L1) binds PD-1 on T cells, promoting immune escape. Cancer-associated fibroblasts (CAFs) secrete WNT2, which blocks DC differentiation, and maintain immunosuppressive stroma through YAP1 activation. Echinacoside reduces PD-L1 expression and enhances T cell infiltration. Inhibition of PTPN3 activates T cell function under hypoxic conditions. NTRK1 signaling suppresses T cell responses; its inhibition sensitizes tumors to ICIs. Combination therapies, such as anlotinib with anti-PD-1, restore immune activation by enhancing DC function and reversing immune resistance. This figure was created using Adobe Illustrator 2024.

#### 3.1.2 Ferroptosis and Tryptophan Metabolism

The immune escape mechanisms of cold tumors significantly restrict the efficacy of immunotherapy, particularly in chemotherapy-resistant cancers such as breast cancer. Recent studies have highlighted the pivotal roles of ferroptosis and tryptophan metabolism pathways within the tumor immune microenvironment. Ferroptosis occurring in tumor-infiltrating neutrophils—an extrinsic microenvironmental factor—suppresses CD8^+^ T cell proliferation and cytotoxicity through the secretion of prostaglandin E2 (PGE2), indoleamine 2,3-dioxygenase (IDO), and oxidized lipids, and is accompanied by the accumulation of IL1β^+^ CXCL3^+^ CD4^+^ T cells. This extrinsic regulatory axis promotes immune resistance by enhancing neutrophil ferroptosis via IL1β/IL1R1/NF-κB-mediated MOAT1 expression. Targeting these immunosuppressive signals, especially IL1β^+^CD4^+^ T cells or IL1R1^+^ neutrophils, can disrupt immune escape, strengthen antitumor immunity, and overcome chemotherapy resistance [118]. Nevertheless, clinical translation of ferroptosis-targeted therapies faces substantial obstacles. Current agents such as sorafenib and sulfasalazine are linked to safety concerns, including hepatic dysfunction and gastrointestinal toxicity [119]. Combining clinically approved ferroptosis inducers with photodynamic therapy shows promise but is complicated by unpredictable drug-related adverse effects, which hinder efficacy assessment [119]. Although ferroptosis combined with immunotherapy holds therapeutic potential, the precise mechanisms of interaction with the immune system remain unclear. Immune cells within the tumor microenvironment are susceptible to ferroptosis, yet the balance between ferroptosis in cancer cells and immune cells remains poorly understood, further limiting clinical application [120]. Due to the high heterogeneity of the tumor microenvironment, ferroptosis exerts complex and context-dependent effects on tumor immunity, leading to unpredictable clinical trial outcomes. The optimal timing for ferroptosis induction or inhibition and the specific target cell populations have yet to be defined, while varying responses among immune cell subsets further complicate safety and efficacy evaluations [121].

Additionally, tryptophan metabolism facilitates immune escape through tumor-intrinsic upregulation of IDO and tryptophan 2,3-dioxygenase (TDO) enzymes. As an extrinsic immunosuppressive mechanism, L-kynurenine activates the aryl hydrocarbon receptor (AHR) pathway, promoting the generation of Tregs and tolerogenic myeloid cells that inhibit CD8^+^ T cell function. Inhibition of AHR can reverse this immunosuppressive effect, thereby enhancing immune responses, particularly when combined with immune checkpoint inhibitors, resulting in significantly improved efficacy. Targeting the intrinsic IDO/TDO-driven activation of the AHR pathway alongside immune checkpoint blockade represents a promising strategy to overcome extrinsic immune suppression and optimize therapeutic outcomes [122].

#### 3.1.3 Adenosine Pathway

Adenosine, produced by Cluster of Differentiation 39 (CD39) and Cluster of Differentiation (CD73) in the tumor microenvironment as an extrinsic immunosuppressive mechanism, binds to the Adenosine A1 Receptor, Adenosine A2A Receptor, Adenosine A2B Receptor and Adenosine A3 Receptor receptors, thereby inhibiting T cell activity and promoting Treg accumulation. Consequently, targeting the adenosine signaling pathway has emerged as a promising immunotherapy strategy, particularly for cold tumors. CD39/CD73 inhibitors and adenosine receptor antagonists are currently under clinical evaluation to reverse immunosuppression in these tumors. Studies have shown that anti-CD39 antibodies restore immune cell function by blocking the extracellular conversion of ATP to adenosine, thereby enhancing anti-tumor responses. When combined with immune checkpoint inhibitors (e.g., PD-1 blockade), anti-CD39 therapy further improves anti-tumor efficacy [123]. Moreover, radiotherapy-induced ATP release is hydrolyzed by CD39 and CD73 within the tumor microenvironment, producing adenosine that suppresses anti-tumor immunity. Targeting CD39/CD73 and adenosine receptors with agents such as anti-CD73 antibodies and small molecule antagonists can relieve this immunosuppression, augment immune responses, and enhance the therapeutic efficacy of radiotherapy [124]. CD73, a key enzyme in adenosine production driven by extrinsic microenvironmental regulation, has gained increasing recognition for its immunosuppressive role. AB680 (quemliclustat), a novel small-molecule CD73 inhibitor, effectively blocks adenosine generation and restores T cell proliferation and cytokine secretion. Preclinical studies demonstrate that AB680, combined with immune checkpoint inhibitors (e.g., anti-PD-1), significantly enhances T cell activity and anti-tumor effects, especially in melanoma models [125]. However, clinical application of AB680 faces challenges, including heterogeneity in patient responses due to varying dependence on CD73, immune-related toxicities potentially triggered by CD73 inhibition in normal tissues, and the risk of drug resistance caused by upregulation of CD73 when combined with immune checkpoint blockade, radiotherapy, or chemotherapy [125]. Additionally, extrinsic interactions between adenosine signaling and immunosuppressive cells, such as plasmacytoid dendritic cells (pDCs), are noteworthy. Hypoxia-induced adenosine enhances pDC recruitment to tumors via A1 receptors, suppressing CD8^+^ T cell proliferation and cytotoxicity [126]. Depletion of pDCs or administration of A1 receptor antagonists significantly improves anti-tumor immunity, offering novel therapeutic insights for cold tumors like hepatocellular carcinoma [126]. Therapeutic strategies targeting the adenosine pathway have shown antitumor potential in various preclinical and clinical studies. Nonetheless, their applicability and efficacy remain limited by dependence on specific target expression in myeloid cells, adverse effects such as fatigue and nausea, and incomplete understanding of immune microenvironment regulation [123]. These factors restrict their broader clinical utility and predictability of therapeutic outcomes.

### 3.2 Remodeling Immune Cell Infiltration

Cold tumor treatment is challenged by insufficient immune cell infiltration, primarily due to extrinsic microenvironmental factors such as abnormal vasculature and decreased chemokine expression. Therefore, remodeling the tumor immune microenvironment has become a critical therapeutic strategy. Chemokine therapy effectively promotes the infiltration of immune cells—particularly CD8^+^ T cells—into the tumor, thereby enhancing the anti-tumor immune response. Epigenetic regulators such as EZH2 and Sin3B further influence chemokine secretion and immune cell recruitment, improving immunotherapy efficacy. Oncolytic virotherapy contributes by lysing tumor cells and modulating tumor-associated macrophage function, which not only boosts immune cell infiltration but also triggers inflammatory responses, fostering an anti-tumor immune environment. When combined with anti-VEGF therapy, which normalizes tumor vasculature and alleviates hypoxia, these approaches synergistically enhance immune cell infiltration and strengthen immune responses. Collectively, these strategies remodel the cold tumor microenvironment, facilitating effective immune cell infiltration and offering promising interventions for cold tumor treatment.

#### 3.2.1 Chemokine Therapy

The senescence-associated secretory phenotype (SASP) directly regulates the recruitment and function of immune cells within the TME by releasing various soluble mediators, including chemokines, cytokines, and growth factors, thus serving as a critical link between cellular senescence and anti-tumor immunity [127]. Recent therapeutic strategies targeting immunosuppressive cold tumors, such as PDAC, have focused on remodeling the TME by modulating the SASP and chemokine networks. For instance, Enhancer of Zeste Homolog 2 inhibitors (EZH2 inhibitors) can alleviate intrinsic tumor resistance by epigenetically derepressing pro-inflammatory SASP genes, thereby increasing the production of C-X-C Motif Chemokine Ligands 9 and 10 (CXCL9 and CXCL10), which promote effector CD8^+^ T-cell infiltration [127]. This chemokine-mediated immune remodeling has been validated across multiple tumor models. For example, Interleukin-15 (IL-15) enhances dendritic cell–T cell interactions by upregulating CXCR3 ligand expression, while deletion of the epigenetic factor Sin3B significantly improves responsiveness to PD-1 blockade through activation of the CXCL9/10–CXCR3 axis [128,129]. Moreover, targeting hypoxia-induced extrinsic suppression mediated by HIF-1α and its upstream regulator Baculoviral IAP Repeat Containing 2 (BIRC2) restores immune infiltration by reactivating CXCL9 and CXCL10 expression [130]. In breast cancer models, histone deacetylase inhibitors increase CXCL9/10 secretion via the NF-κB pathway, facilitating recruitment of CXCR3^+^ CD4^+^ T cells [131], whereas dual antagonists of Chemokine Receptors 2 and 5 (CCR2/5) combined with radiotherapy block recruitment of immunosuppressive cells and enhance T-cell function in PDAC [132]. Studies in other cancers reveal diverse chemokine regulatory mechanisms: Yes-Associated Protein 1 (YAP1) drives intrinsic tumor resistance by suppressing immune infiltration through IL-6/STAT3 signaling in bladder uroepithelial carcinoma [133]; MDSCs limit CD8^+^ T-cell activity via CCR5 in melanoma [134]; and the IL-6/CCL2 pathway inhibits NK cell function in head and neck squamous carcinoma [135]. In hepatocellular carcinoma, B-Cell Lymphoma 6 (BCL6) impairs T cell infiltration by repressing inflammatory chemokines [136]. Innovative therapies, such as engineered Granulocyte-Macrophage Colony-Stimulating Factor/Interleukin-12 (GM-CSF/IL-12) combinations, may overcome immune checkpoint inhibitor resistance by modulating tumor-associated macrophage polarization and dendritic cell activation [137], while apigenin inhibits tumor necrosis factor-alpha (TNFα)-driven CCL2 release, blocking immunosuppressive cell recruitment [138]. Collectively, these findings highlight the therapeutic potential of remodeling the immune microenvironment by targeting the SASP-associated chemokine network, offering a multi-targeted approach to convert cold tumors into immunologically active hot tumors.

#### 3.2.2 Oncolytic Virotherapy

Oncolytic virotherapy has demonstrated significant potential in overcoming extrinsic microenvironmental suppression in cold tumors, particularly in non-small cell lung cancer, by inducing inflammation and enhancing infiltration of cytotoxic immune cells. Low-pathogenicity influenza A virus (IAV) promotes M1 macrophage polarization through tumor cell lysis and modulation of TAM function, thereby augmenting cytotoxic immune cell infiltration. When combined with novel B7-H3 ICIs, this approach significantly enhances immune cell infiltration and anti-tumor efficacy, outperforming traditional α-PD-1 antibody monotherapy [139]. The oncolytic virus Ad5/3-d24-E2F-hTNFa-IRES-hIL2 (TILT-123 or OAd.TNFa-IL2) remodels the tumor microenvironment by inducing the release of danger-associated molecular patterns (DAMPs) and pathogen-associated molecular patterns (PAMPs), activating Absent in Melanoma 2 inflammasomes, and fostering an immune-stimulatory, anti-tumor milieu. This therapy not only lysates tumor cells but also counters extrinsic suppression by increasing cytotoxic T cell and macrophage infiltration, thereby enhancing anti-tumor immunity [140]. Moreover, oncolytic viruses can promote the formation of tertiary lymphoid structures (TLS), further boosting immune responses and the infiltration of cytotoxic T cells and dendritic cells. Combination with immune checkpoint inhibitors markedly improves these effects [141]. Systemic delivery of oncolytic viruses in glioblastoma (GBM) has been challenging; however, encapsulating CXCL11-modified oncolytic adenovirus with tannic acid and Fe^3+^ has extended circulation time and reshaped the tumor immune microenvironment effectively [142]. Despite these advances, clinical application of oncolytic virus therapy (OVT) faces dual challenges of efficacy and safety. Although combining oncolytic viruses with conventional therapies can improve objective response rates, long-term survival benefits remain limited and are often accompanied by severe adverse events [143]. A critical obstacle is insufficient tumor targeting, as wild-type viruses lack specificity, necessitating genetic modifications to enhance tumor tropism [144]. Viral spread is hindered by tumor penetration barriers and host immune clearance, while administration routes—intratumoral or intravenous—pose risks of uneven distribution or off-target infection [145]. Although genetic engineering can boost antitumor activity, cytokine expression may cause systemic toxicity [144]. Additionally, biosafety concerns and the risk of environmental viral transmission require thorough evaluation [145]. Collectively, these factors constrain the clinical translation of OVT.

#### 3.2.3 Vascular Normalization

The combination of anti-VEGF therapy and immunotherapy has emerged as an effective strategy for remodeling the TME and enhancing immune responses. Studies have shown that low ATM scores are closely associated with plasma cell activation pathways. The ATM model not only predicts the efficacy of immune checkpoint blockade therapies but also correlates with increased sensitivity to anti-VEGF treatments [146]. In osimertinib-resistant NSCLC, combining the anti-angiogenic agent Anlotinib significantly enhances T-cell infiltration, particularly boosting the proportion of cytotoxic T cells, thereby improving anti-tumor immunity [147]. However, osimertinib-based combination antiangiogenic therapy relies on the immune-enhancing effects of vascular normalization within the TME and faces limitations due to molecular heterogeneity of resistance mechanisms, difficulties in tissue rebiopsy for target accessibility, and limited predictive value [147]. Hypoxia and other extrinsic immunosuppressive factors in the TME remain major barriers in treating cold tumors. Research indicates that ultrasound microbubble cavitation induced by low mechanical index ultrasound improves tumor perfusion and oxygenation, promoting vascular normalization and alleviating immune suppression [148]. Concurrently, loss of Rap1B enhances CD8^+^ T-cell infiltration, suggesting that targeting Rap1B could represent a novel strategy to overcome tumor immune suppression [149]. While combined antiangiogenic and immunotherapy approaches show promise, there is a pressing need for novel, more targeted agents. Although targeting Rap1B offers potential to improve vascular function and immunotherapy efficacy, its underlying molecular mechanisms remain unclear, and inhibitor development poses challenges [149]. Garcinia alleviates extrinsic microenvironmental suppression by reducing hypoxic stress through inhibition of HIF-1α and VEGF, enhancing immune cell infiltration, and augmenting anti-tumor immunity when combined with anti-PD-L1 treatment [150]. Moreover, TU2218, a dual inhibitor of TGFβ and VEGF, mitigates extrinsic immune suppression by restoring cytotoxic T-cell activity and reversing VEGF-induced endothelial dysfunction, thereby enhancing immunotherapy effectiveness [151]. Finally, Zinc Finger and BTB Domain-Containing Protein 46 (ZBTB46) plays a critical role in regulating angiogenesis and the extrinsic immune environment; its upregulation promotes vascular normalization and immune restoration, significantly improving therapeutic outcomes when combined with anti-PD-1 therapy [152]. Collectively, these studies demonstrate that integrating anti-VEGF therapy with immunotherapy—particularly through vascular normalization and immune cell remodeling—offers a promising intervention for cold tumors.

### 3.3 Metabolic Reprogramming Interventions

Metabolic reprogramming within the tumor microenvironment profoundly influences immune cell function by dynamically altering their metabolic phenotypes. Tumor cells increase exogenous fatty acid uptake via elevated expression of low-density lipoprotein receptors, fatty acid translocases, and fatty acid-binding proteins, which not only suppress immune cell activity but also promote tumor proliferation and epithelial-mesenchymal transition [153]. Key enzymes involved in *de novo* fatty acid synthesis, such as ATP-citrate lyase and acetyl-CoA carboxylase, are upregulated, enhancing tumor energy supply and mediating immune escape through metabolic intermediates. Concurrently, fatty acid oxidation (FAO) supplants glucose metabolism as the dominant energy source, further driving immunosuppressive phenotypes [153]. Metabolic reprogramming exerts bidirectional regulation on macrophage function: early pro-inflammatory signals polarize macrophages to the M1 phenotype, reliant on glycolysis and the pentose phosphate pathway, with accumulation of tricarboxylic acid (TCA) cycle intermediates promoting antitumor effects via mitochondrial reactive oxygen species and nitric oxide production. In contrast, later anti-inflammatory signals induce M2 polarization characterized by oxidative phosphorylation and glutamine metabolism, accompanied by a shift in arginine metabolism toward the urea cycle, which supports tumor proliferation and metastasis [154]. In NSCLC, tumor-associated macrophages exhibit high expression of the MTHFD1 gene, which drives immunosuppressive differentiation through metabolic reprogramming, while also promoting endothelial proliferation and angiogenesis, thereby exacerbating immune escape and resistance to therapy [155]. T-cell function is similarly modulated by metabolic remodeling: PD-1 signaling inhibits glycolysis and amino acid metabolism, augments FAO, and upregulates carnitine palmitoyl transferase 1A (CPT1A), collectively blocking effector T-cell differentiation and facilitating metabolic adaptation [156]. HIF-1α regulates fatty acid oxidation in Tregs, supporting their immunosuppressive roles within hypoxic tumor niches [157]. Targeting metabolic enzymes offers promising therapeutic avenues: inhibition of lactate dehydrogenase A (LDHA) reduces lactate accumulation, reverses CD8+ T-cell dysfunction, and remodels the high-glucose, low-lactate microenvironment [158]; glutaminase (GLS) inhibitors disrupt tumor metabolic dependence, synergize with immune checkpoint blockade, and enhance CAR-T cell infiltration and efficacy [159]. Furthermore, metabolic reprogramming driven by the c-MET signaling pathway activates CAFs and MDSCs, promoting immune escape through effector immune cell inhibition; targeting c-MET alongside immunotherapy substantially improves outcomes [160]. The HIF-1α/Stearoyl-CoA Desaturase 1 (SCD1) axis-mediated accumulation of lipid droplets and HVEM-regulated aerobic glycolytic reprogramming in T cells further highlight the complex, multidimensional potential of metabolic interventions in cold tumor therapy [161,162].

Metabolic reprogramming strategies targeting LDHA and GLS have emerged as crucial approaches in treating cold tumors. Lactate accumulation within the tumor microenvironment impairs CD8^+^ T-cell effector functions while enhancing the immunosuppressive activity of regulatory T cells, thereby facilitating extrinsic immune evasion [158]. Inhibiting LDHA reduces lactate buildup, restoring a high-glucose, low-lactate microenvironment that promotes CD8^+^ T-cell activation and strengthens anti-tumor immunity [158]. Additionally, glutamine metabolism, regulated by GLS activity, plays a pivotal role in immune cell function. Targeting glutamine transporters disrupts the metabolic dependencies of tumor cells and, in combination with immune checkpoint inhibitors, significantly enhances anti-tumor immune responses [159]. The GLS inhibitor CB-839 effectively remodels the tumor metabolic landscape, improving CAR-T cell infiltration and boosting the overall efficacy of immunotherapy [159].

### 3.4 Personalized Neoantigen Vaccines and Cell Therapy

Antigen vaccines and cell therapies hold promising potential for treating cold tumors. Neoantigen vaccines activate T cells and, when combined with modalities such as photodynamic therapy or viral vectors, effectively improve the tumor immune microenvironment. Dendritic cell vaccines, particularly when paired with adoptive cell transfer, enhance T-cell infiltration into tumors, helping to overcome extrinsic barriers to immune activation. Notably, the combination of T-cell receptor-engineered T cells (TCR-T) and NK cells represents a novel, personalized therapeutic strategy for cold tumors.

#### 3.4.1 Neoantigen Vaccine Design

Recent advances demonstrate that viral vector-based platforms, such as those utilizing lymphocytic choriomeningitis virus (LCMV), can effectively break immune tolerance and activate CD8^+^ T cells, thereby converting immunologically cold tumors into hot, inflamed microenvironments [163]. Innovative delivery systems further enhance vaccine efficacy—for example, acidic and photosensitive DC-based neoantigen nanovaccines synergize with photodynamic therapy to promote tumor antigen release and amplify immune responses in hepatocellular carcinoma. DC vaccines loaded with tumor lysates have been shown to expand neoantigen-reactive T cells and improve adoptive cell transfer (ACT)-mediated tumor infiltration [164]. Mechanistic studies reveal that while CD4^+^ T cells contribute to protective immunity, optimal antitumor effects depend critically on CD8^+^ T cell activity within the bone marrow niche [165]. Epitope optimization strategies, such as NitraTh-based designs, overcome limitations of conventional CD4^+^ T cell epitope prediction and remodel the immunosuppressive microenvironment by reprogramming macrophages and reducing prostaglandin E2 (PGE2), thereby inhibiting hepatocellular carcinoma progression [166]. Additionally, virus-like silica vaccines with spike-like structures co-deliver hepatocellular carcinoma neoantigens and TLR9 agonists, effectively enhancing CD8^+^ T cell responses against both primary and metastatic tumors [167]. However, platforms based on shared neoantigens face challenges from HLA restriction and tumor immune editing, which may promote immune escape [168]. Similarly, neoantigen peptide vaccines show immunogenicity in NSCLC, but HLA dependency and patient-to-patient variability complicate precision application [169]. Fundamental limitations remain, including low immunogenicity in tumors with few mutations, heterogeneous antigen expression causing immune evasion, dysfunctional antigen presentation, and difficulties in scalable personalized manufacturing [170]. Despite these hurdles, neoantigen vaccines continue to hold significant promise in cancer immunotherapy by reshaping cold tumor microenvironments through targeted T cell activation.

#### 3.4.2 Multi-Omics Optimization Strategies for Cell Therapy

Cellular therapies have demonstrated diverse potentials and challenges in tumor treatment, particularly for cold tumors. TCR-T therapies are emerging as promising approaches, especially γδ T cells, which recognize lipid antigens and exhibit robust immune responses due to their antigen-processing-independent and HLA-unrestricted properties. Although γδ T cells have not yet entered clinical trials, they hold great potential in cold tumor therapy [171]. Combining TCR-T and chimeric antigen receptor (CAR) T-cell therapies shows synergistic effects by targeting distinct tumor antigens—TCR-T cells primarily recognize intracellular antigens presented by MHC, whereas CAR-T cells target surface antigens, resulting in improved therapeutic efficacy [171]. Despite challenges posed by the loss of major histocompatibility complex (MHC) expression, strategies to enhance MHC levels and neoantigen recognition remain promising; for instance, NY-ESO-1-specific TCR-T cells demonstrated significant clinical efficacy in synovial sarcoma [172]. TILs outperform conventional immune cells by recognizing multiple antigens, yet their efficacy is limited by tumor microenvironmental suppression and antigenic mutations. Multi-omics approaches are being used to optimize TIL function by enhancing antigen recognition and alleviating immune suppression [173]. NK cell therapies benefit from genetic engineering that improves their migration, expansion, and persistence, such as modifications to chemokine receptors and CAR constructs. Engineered NKG2D.ζ-NK cells have shown the capacity to overcome immunosuppressive tumor microenvironments, offering a novel avenue for cold tumor treatment [172]. CAR-T cells have demonstrated the ability to cross the blood-brain barrier for treating central nervous system lymphomas, but high relapse rates, cytokine release syndrome, and neurotoxicity risks limit broader application, necessitating further optimization of CAR designs and combination regimens [174]. Autologous whole-cell vaccines offer a favorable safety profile and potential survival benefits as adjuvant therapies in solid tumors, though heterogeneity in clinical trial protocols and patient populations complicates efficacy assessments [175]. CAR-T therapies in solid tumors face hurdles including microenvironmental inhibition, hypoxic and acidic conditions, antigen heterogeneity, and off-target toxicity [176]. Similarly, TCR-T therapies are constrained by off-target effects, limited target antigen selection, and tumor microenvironment-mediated suppression [177]. Non-transgenic NK cell therapies have improved remission rates in cancers like hepatocellular carcinoma, but factors such as cell source variability, combination treatments, and cytokine use (e.g., IL-2, which may inadvertently stimulate regulatory T cells) impact their efficacy [178]. Overall, while cellular therapies offer significant promise in cold tumor treatment, overcoming microenvironmental barriers, managing toxicity, and establishing standardized protocols remain essential for their broader clinical translation.

### 3.5 Epigenetic Regulation

Epigenetic modifications play a crucial role in regulating immune evasion across various cancer types through mechanisms such as DNA methylation and histone modifications. In recurrent lung adenocarcinoma, hypomethylation of the TEAD1 binding site leads to aberrant activation of the PRAME gene, which in turn promotes the upregulation of epithelial-mesenchymal transition (EMT)-related genes and enhances metastatic potential [98]. Conversely, in NSCLC and melanoma, genomic demethylation suppresses the transcription of immunoregulatory genes, thereby weakening the immune-activating effects associated with a high mutational load [179]. In gastrointestinal cancers, DNA methyltransferases DNMT1 and DNMT3B mediate methylation of the CIITA promoter, silencing its expression and consequently blocking HLA-DR induction, which limits tumor suppressor functions of CD4^+^ lymphocytes [180]. Histone modifications similarly influence immune evasion: overexpression of histone deacetylases (HDACs) in pancreatic cancer represses tumor suppressor genes and dampens anti-tumor immune responses, while HDAC inhibitors can restore T cell activation and increase tumor immunogenicity [181]. In breast cancer, reduced histone acetylation alongside inhibitory histone marks promotes immune escape by upregulating PD-L1 expression; this effect is further amplified by hypomethylation of immune checkpoint gene promoters coupled with decreased H3K9me3 and H3K27me3 modifications [182,183]. The HTLV-1 virus in adult T-cell leukemia/lymphoma (ATL) induces aberrant epigenetic changes through viral proteins Tax and HTLV-1 bZIP factor (HBZ), fostering a regulatory T cell-like immunosuppressive phenotype [184]. Furthermore, the combination of the HDAC inhibitor panobinostat and the BRD4 inhibitor JQ1 in metastatic colorectal cancer modulates glycolysis and inhibits PD-L1 expression, enhancing CD8^+^ T-cell function via targeting of low-density lipoprotein-related protein 1 (LRP-1) [185]. Collectively, these studies highlight that epigenetic modifications intricately regulate the tumor immune microenvironment through multiple pathways, offering a theoretical foundation for novel targeted interventions.

More importantly, epigenetic regulation offers promising new strategies for the treatment of cold tumors. Histone deacetylase (HDAC) inhibitors have been shown to enhance interferon signaling and promote immune cell infiltration, thereby reversing the extrinsic immunosuppressive conditions within the tumor microenvironment and improving responsiveness in tumors that are resistant to immunotherapy. Beyond histone modifications, RNA methylation—particularly m6A modifications—also plays a critical role in shaping antitumor immune responses. m6A modifications influence immunotherapy outcomes by modulating the expression of immune checkpoint genes within tumor cells and altering patterns of immune cell infiltration, thereby affecting both intrinsic resistance mechanisms and extrinsic immune suppression. The dynamic regulation of m6A by writer (e.g., METTL3) and eraser (e.g., FTO, ALKBH5) enzymes further fine-tunes the expression of immune-related genes, ultimately improving the effectiveness of immune responses. These findings underscore the therapeutic potential of epigenetic interventions to reprogram cold tumor microenvironments and sensitize them to immunotherapeutic strategies.

#### 3.5.1 HDAC Inhibitors Activate Interferon Signals

HDAC inhibitors play a pivotal role in tumor immunotherapy by modulating the interferon (IFN) signaling pathway, exerting multi-level effects on both intrinsic and extrinsic immune resistance. For instance, the combination of the class I HDAC inhibitor entinostat with the immunocytokine NHS-IL12 effectively restores MHC class I expression and IFN-γ signaling in tumor cells, thereby targeting intrinsic immune resistance. This combination also reprograms the immunosuppressive tumor microenvironment by activating the JAK/STAT and IFN-γ pathways, stimulating M1 macrophages and antigen-presenting cells, and promoting CD8^+^ T-cell infiltration to alleviate extrinsic immunosuppression [186]. However, therapeutic efficacy varies across tumor models; in the IFN-γ-resistant RVP3 model, this regimen shows limited enhancement of CD8^+^ tumor-infiltrating lymphocytes and pro-inflammatory cytokines, indicating an incomplete reversal of tumor-intrinsic immune escape mechanisms [186]. Similarly, in ovarian cancer, epigenetic modulators such as DNA methyltransferase (DNMT) inhibitors and HDAC6 inhibitors enhance immune cell infiltration by restoring type I interferon signaling and mitigating extrinsic immune suppression. Yet, these interventions concurrently upregulate PD-L1 expression and increase T-cell PD-1 levels, potentially inducing an immunosuppressive feedback loop that limits long-term therapeutic benefit [187]. These findings suggest that while HDAC inhibitors hold substantial promise in activating IFN pathways and reprogramming the tumor immune microenvironment, their clinical efficacy remains constrained by tumor heterogeneity, immune evasion strategies, and compensatory immunosuppressive mechanisms. Thus, optimizing their application across different tumor contexts and in combination with immune checkpoint blockade may be key to enhancing their therapeutic impact.

#### 3.5.2 m6A Modification Regulates Immunotherapeutic Response

N6-methyladenosine (m6A) modification plays a crucial regulatory role within the TME, profoundly influencing immune responses and the efficacy of immunotherapy in cold tumors. Emerging evidence suggests that m6A regulators modulate immune infiltration through extrinsic mechanisms and govern immune evasion via intrinsic alterations in tumor gene expression, thereby shaping tumor immunogenicity and therapeutic responsiveness. For example, the m6A writer METTL3 enhances anti-tumor immunity by promoting infiltration of CD8^+^ T cells and NK cells, effectively reversing extrinsic immunosuppressive conditions [188]. Conversely, the m6A erasers FTO and ALKBH5 contribute to intrinsic immune escape by modulating the expression of immune checkpoint molecules such as PD-1 and PD-L1 [188]. In low-grade glioma, aberrant upregulation of m6A regulators correlates with alterations in the immune microenvironment, reflecting extrinsic immunological remodeling, while the elevated expression of PD-L1 and PD-1 driven by FTO and ZCCHC4 indicates intrinsic resistance to immune surveillance [189]. Similarly, in lung adenocarcinoma, distinct m6A modification patterns correspond with specific immune phenotypes that reflect both intrinsic transcriptional programs and the extent of extrinsic immune activation, with high m6A scores associated with increased sensitivity to immunotherapy [190]. Therapeutically, targeting m6A regulators—such as through inhibition of METTL3 or FTO—disrupts tumor-intrinsic immune resistance and reshapes extrinsic immunosuppressive networks, thereby enhancing the efficacy of immune checkpoint inhibitors in cold tumor settings [191,192]. These findings underscore the dual regulatory potential of m6A modification and highlight its value as a promising target for overcoming immunotherapy resistance in cold tumors.

### 3.6 Clinical Translation Efforts

The clinical translation of tumor treatment strategies increasingly relies on diverse and innovative preclinical models to optimize precision medicine and improve immunotherapy outcomes. Among these, patient-derived organoids (PDOs) have demonstrated significant value. In esophageal squamous cell carcinoma, PDOs recapitulate tumor characteristics with a 61.8% establishment rate, and their chemotherapy sensitivity profiles closely correlate with clinical outcomes. Notably, patients in the therapy-sensitive group exhibit significantly prolonged progression-free survival, underscoring the potential of PDOs to guide individualized treatment [193]. However, in immunotherapy-refractory malignancies such as pancreatic ductal adenocarcinoma (PDA), PDO models incorporating T cells have enabled more accurate reconstruction of the immunosuppressive tumor microenvironment. These co-culture systems support high-throughput screening and have identified epigenetic inhibitors, such as ITF2357 and I-BET151, that synergize with anti-PD-1 therapy to enhance immune activation, reverse immunosuppression, and improve effector T cell function [194]. Humanized mouse models further enhance translational relevance by integrating components of the human immune system. Anti-PD-1 therapy in these models has been shown to amplify human T cell antitumor responses while reducing regulatory T cells and myeloid-derived suppressor cells, validating their utility in immunotherapy research [195]. Additionally, vascularized and immune-infiltrated patient-derived tumor (PDT) models reflect primary tumor heterogeneity and facilitate rapid assessment of responses to anti-angiogenic and chemotherapeutic agents, as well as modulation of key markers such as Vascular Endothelial Growth Factor-A (VEGF-A), Lymphocyte Activation Gene 3 (LAG-3), and B and T Lymphocyte Attenuator (BTLA), supporting personalized approaches in lung cancer [196]. Despite their advantages, PDOs inherently lack immune and vascular components, limiting their utility for immunotherapy evaluation. Moreover, variability in model construction and the absence of standardized evaluation protocols pose significant barriers to clinical translation [197,198]. Humanized patient-derived xenograft (PDX) models offer complementary advantages. In ovarian clear cell carcinoma, these models demonstrate superior therapeutic responses to PI3K inhibitors compared to conventional PDXs, and show synergistic trends when combined with anti-PD-1 therapy, emphasizing the influence of immune context on therapeutic efficacy [199]. The MISTRG6 humanized mouse model, which incorporates patient-derived hematopoietic stem and progenitor cells, achieves comprehensive genetic matching of the tumor microenvironment, enabling the simulation of innate and adaptive immune interactions, including VEGF-A expression [200]. In NSCLC, PDX models established in NOD/Shi-scid/IL-2Rγnull mice exhibit distinct immune infiltration profiles between immunologically “hot” and “cold” tumors. Digital image analysis confirms histological fidelity, facilitating the evaluation of immuno-oncology strategies and biomarker discovery [201]. Sequential PDX engraftment—initially in immunodeficient mice and subsequently in humanized hosts—has been shown to reduce immune-mediated toxicity while preserving long-term tumor growth and microenvironmental stability. In melanoma, this approach revealed superior anti-tumor efficacy of rigosertib over anti-PD-1 therapy, including improved modulation of CD8^+^/CD4^+^ T cell ratios, highlighting the need for optimized immune-editing models to accurately assess therapeutic responses [202]. Nonetheless, humanized PDX models remain constrained by long development timelines, high costs, incomplete innate immune system reconstitution, immune architectural deficiencies, and an inability to fully capture tumor heterogeneity and clonal evolution, all of which limit their clinical applicability [203,204]. Despite these challenges, the integrative use of PDOs, humanized mice, and PDT models is accelerating advances in precision immunotherapy, providing robust platforms for tailoring cancer treatment to individual patients (Table 2).

**Table 2:** Therapeutic intervention strategies

	Intervention category	Target/Strategy	Mechanism of action	References
Targeted immunosuppressive signals	Immune checkpoint combination therapy	GAD1/GABA signaling pathway	Inhibit GABA-mediated β-catenin pathway activation, promote CD8^+^ T cell infiltration	[103]
		DKK1	Regulate TAM phenotype, enhance PD-1 blockade effect	[104]
		MMP9	Restore T cell function in CTNNB1 mutant HCC	[105]
		WNT2-CAFs	Restore DC differentiation, enhance immune response	[106]
		LRP1/NOTCH	Reverse M2 macrophage polarization, overcome ICI resistance	[107]
		YAP1-CAFs	Convert CAF phenotype, enhance immune response	[108]
		PABPC1L/IDO1	Reduce Treg infiltration, improve T cell function	[109]
		FTSJ3	Activate the type I interferon pathway	[110]
		exoPD-L1	Block exosome-mediated immune evasion	[111]
	Ferroptosis and metabolism	IL1β/IL1R1/NF-κB	Target neutrophil ferroptosis, reverse immune resistance	[118]
		IDO/TDO-AHR pathway	Inhibit Kyn-AHR pathway, restore CD8^+^ T cell function	[122]
	Adenosine pathway	CD39/CD73 inhibitors	Blocks ATP-to-adenosine conversion, restoring T-cell function	[123]
		AB680 (CD73 inhibitor)	Inhibits adenosine receptor signaling, enhances anti-PD-1 efficacy by inhibiting adenosine production	[125]
		A1 receptor antagonists	Reduces pDC-mediated CD8^+^ T-cell suppression	[126]
Immune cell remodeling	Chemokine therapy	EZH2 inhibitors	Upregulates CXCL9/10 to recruit CD8^+^ T cells	[127]
		Sin3B deletion	Activates CXCL9/10-CXCR3 axis to enhance PD-1 sensitivity	[128,129]
		HIF-1α/BIRC2 inhibition	Restores CXCL9/10 expression under hypoxia	[130]
		HDAC inhibitors	Promotes CXCR3^+^CD4^+^ T cell recruitment via NF-κB	[131]
	Oncolytic virus therapy	IAV + B7-H3 ICIs	Polarizes M1 macrophages and enhances cytotoxic T-cell infiltration	[139]
		OAd.TNFa-IL2	Induces DAMPs/PAMPs to activate AIM2 inflammasomes	[140]
	Vascular normalization	Anti-VEGF + ICIs	Improves T-cell infiltration via vascular remodeling	[146]
		Anlotinib	Reverses osimertinib resistance by increasing cytotoxic T cells	[147]
		ZBTB46 overexpression	Promotes vascular normalization and synergizes with anti-PD-1	[152]
Metabolic reprogramming		LDHA inhibitors	Reduces lactate accumulation, restoring CD8^+^ T cell function	[158]
		CB-839 (GLS inhibitor)	Disrupts glutamine metabolism to enhance CAR-T efficacy	[159]
Personalized therapy	Neoantigen vaccine	LCMV-based vaccines	Breaks immune tolerance via CD8^+^ T-cell activation	[163]
		DC nanovaccines + photodynamic therapy	Enhances antigen release and T-cell infiltration	[164]
	Cell therapy	γδ TCR-T cells	Recognizes lipid antigens independent of HLA	[171]
		NY-ESO-1 TCR-T	Targets MHC-presented neoantigens in synoviocyte sarcoma	[172]
Epigenetic regulation	HDAC inhibitors	Entinostat/NHS-IL12	Activate IFN-γ signaling, enhance CD8^+^ T/M1 macrophage infiltration	[186]
	m6A modification	METTL3/FTO targeting	Regulate immune checkpoint expression, enhance ICB efficacy	[188–192]
Clinical translation efforts	Organoid models	PDA organoids + ITF2357/I-BET151	Identifies epigenetic inhibitors to enhance anti-PD-1 efficacy	[194]
	Humanized PDX models	MISTRG6 mice	Simulates tumor-immune interactions with matched TME	[200]

Note: GAD1, Glutamate Decarboxylase 1; GABA, γ-aminobutyric acid; DKK1, Dickkopf-1; TAM, Tumor-associated macrophage; PD-1, Programmed Death-1; MMP9, Matrix Metallopeptidase 9; HCC, Hepatocellular carcinoma; WNT2, Wingless-Type MMTV Integration Site Family Member 2; CAFs, Cancer-associated fibroblasts; DC, Dendritic cell; LRP1, Low-Density Lipoprotein Receptor-Related Protein 1; NOTCH, Notch Homolog; ICI, Immune checkpoint inhibitor; YAP1, Yes-associated protein1; PABPC1L, Poly(A) Binding Protein Cytoplasmic 1 Like; IDO1, Indoleamine 2,3-Dioxygenase 1; FTSJ3, FtsJ RNA 2^′^-O-methyltransferase 3; exoPD-L1, exosomal PD-L1; IL1^β^, Interleukin 1 Beta; IL1R1, Interleukin 1 Receptor 1; NF-κB, Nuclear Factor Kappa-B; TDO, Tryptophan 2,3-Dioxygenase; CD39, Cluster of Differentiation 39; CD73, Cluster of Differentiation 73; ATP, Adenosine Triphosphate; EZH2, Enhancer of Zeste Homolog 2; CXCL9/10, Chemokine (C-X-C Motif) Ligand 9/10; CXCR3, C-X-C Motif Chemokine Receptor 3; HIF-1α, Hypoxia-Inducible Factor 1 Alpha; BIRC2, Baculoviral IAP Repeat-Containing Protein 2; HDAC, Histone Deacetylase; IAV, Influenza A virus; ICIs, Immune checkpoint inhibitors; OAd.TNFa-IL2, Oncolytic Adenovirus Expressing TNF-αand IL-2; DAMPs, Damage-Associated Molecular Patterns; PAMPs, Pathogen-Associated Molecular Patterns; AIM2, Absent in Melanoma 2; VEGF, Vascular Endothelial Growth Factor; ZBTB46, Zinc Finger and BTB Domain-Containing Protein 46; LDHA, Lactate dehydrogenase A; GLS, Glutaminase; CAR-T, Chimeric Antigen Receptor T-Cell; LCMV, Lymphocytic Choriomeningitis Virus; DC, Dendritic cell; TCR-T, T-cell receptor engineered T cells; HLA, Human Leukocyte Antigen; NY-ESO-1, New York Esophageal Squamous Cell Carcinoma-1; MHC, Major histocompatibility complex; NHS-IL12, NHS76 Antibody-Human IL-12 Fusion Protein; IFN-γ, Interferon-γ; METTL3, Methyltransferase Like 3; FTO, Fat mass and obesity-associated protein; ICB, Immune checkpoint blockade; PDA, Pancreatic Ductal Adenocarcinoma; ITF2357, Inhibitor of Histone Deacetylase; I-BET151, Inhibitor of BET Protein; MISTRG6, Mouse model with human M-CSF, IL-3, GM-CSF, SIRPα, ThPO, and IL-6 genes; TME, Tumor microenvironment.

## 4 Discussion

### 4.1 The Value and Limitations of Multi-Omics Integration

Multi-omics integration—enabled by the convergence of advanced technological platforms—offers a comprehensive framework for dissecting the tumor immune microenvironment, thereby uncovering critical biological insights and advancing the precision of immunotherapy. The synergistic application of next-generation sequencing (NGS) with immune cell profiling techniques enhances the clinical translation of tumor immunology by providing high-resolution molecular and cellular data [6]. This approach is particularly valuable in tailoring precision oncology strategies for diverse cancer types, including solid tumors and hematologic malignancies. By incorporating genomic, transcriptomic, epigenomic, and spatial omics datasets, multi-omics integration provides an in-depth, systems-level view of tumor biology, capturing the intricate regulatory networks and dynamic cellular states that drive tumor progression and immune evasion. Spatial omics technologies, which preserve the spatial architecture of tissue samples, are instrumental in characterizing cellular neighborhoods and intercellular interactions within the tumor microenvironment. This spatial resolution is essential for understanding extrinsic immunosuppressive mechanisms and for identifying immune exclusion patterns that contribute to the cold tumor phenotype. Furthermore, the integration of multimodal data repositories—such as those supported by the Cancer Moonshot initiative—accelerates discovery in cancer research by promoting cross-platform analytics and collaborative innovation. These resources enable the identification of novel biomarkers, therapeutic targets, and resistance mechanisms, ultimately enhancing the predictive power and therapeutic precision of immuno-oncology [205].

### 4.2 Consensus and Controversies on Immune Escape Mechanisms in Cold Tumors

Genomics reveals that immune escape in cold tumors is largely driven by intrinsic tumor resistance, including gene mutations, epigenetic modifications, and dysregulated expression of immune escape–related genes within tumor cells. Tumor cells often evade immune surveillance through alterations in genes involved in immune checkpoint regulation and antigen presentation. However, the impact of gene mutations varies across tumor types, and how to accurately predict immune escape based on genomic data remains an area requiring further exploration. Moreover, whether the relationship between gene mutations and immune escape is universally applicable remains controversial [8,10]. Transcriptomic studies indicate that cold tumors exhibit both intrinsic tumor resistance, characterized by upregulation of immune escape–related genes, and extrinsic microenvironmental suppression, reflected in elevated expression of immunosuppressive factors that inhibit immune cell activation. In addition, immune cells in cold tumors often exhibit impaired activation due to these extrinsic suppressive signals, further contributing to immune escape. However, the transcriptional regulatory mechanisms underlying different immune escape processes remain unclear. Whether the expression of immune escape–related genes is governed by a single or multiple factors still warrants further investigation [34,35]. Proteomics has revealed increased expression of immune escape–related proteins on the surface of tumor cells, representing intrinsic mechanisms that suppress immune cell activity. At the same time, extrinsic suppression mediated by immunosuppressive cytokines and stromal proteins also plays a critical role in facilitating immune evasion. Nonetheless, protein expression is subject to multifactorial regulation, and current evidence remains insufficient to directly link proteomic profiles with specific immune escape mechanisms. Whether protein–protein interactions within the tumor microenvironment can effectively predict immune escape outcomes remains uncertain [80,81]. Metabolomics has demonstrated that metabolic reprogramming in tumor cells contributes to intrinsic resistance, while the accumulation of acidic metabolites and hypoxia in the tumor microenvironment leads to extrinsic immunosuppression, together impairing immune cell function and promoting immune escape. These hostile metabolic conditions exemplify external barriers that directly suppress immune activity. However, the dual roles of certain metabolic products are not yet fully understood—some may, under specific conditions, actually enhance immune responses. How to balance these complex interactions remains a major challenge in current research [83,85]. Spatial multi-omics analyses reveal substantial spatial heterogeneity in immune cell distribution, suggesting that extrinsic immunosuppression may result from limited spatial proximity between immune and tumor cells. Immune escape in cold tumors is closely linked to spatial inefficiency in immune cell localization. This ineffective spatial distribution constitutes a significant extrinsic barrier to immune activation. However, accurately correlating spatial heterogeneity with underlying immune escape mechanisms remains a technical and interpretive challenge [89,94].

### 4.3 Translational Barriers of Treatment Strategies

Many therapeutic targets—such as GAD1, DAD1, and MMP9—are also expressed in normal tissues, raising concerns about off-tumor toxicity. For example, adenosine pathway inhibitors (e.g., anti-CD39/CD73) may disrupt adenosine homeostasis in the cardiovascular and nervous systems; similarly, targeting GABA signaling could impair neuronal function. In addition, tumor heterogeneity, a hallmark of intrinsic tumor resistance, leads to substantial variability in target expression across patients and even within different regions of the same tumor. For instance, CTNNB1 mutations are only present in a subset of hepatocellular carcinoma, limiting the generalizability of corresponding targeted therapies [103–105,123,125]. Furthermore, combination therapies may inadvertently trigger excessive immune activation. For example, LDHA inhibitors, used in metabolic reprogramming, might impair glycolysis in normal cells, potentially causing toxicity in the gastrointestinal or hematopoietic systems. Inhibition of the IL-1β pathway may suppress anti-infective immunity, raising safety concerns. Oncolytic virus therapies, while designed to remodel the local immune microenvironment and overcome extrinsic immunosuppression, can also induce systemic inflammatory responses, exacerbating immune-related toxicity [118,139,140,158]. More importantly, the translational relevance of current findings is often limited by the use of mouse models, which cannot fully replicate the complexity of the human TME. For example, although oncolytic viruses demonstrate effective infiltration in murine models, dense stromal barriers in human tumors may impede viral delivery and limit efficacy [139,141]. Similarly, the preclinical success of neoantigen vaccines is constrained by the simplified immune architecture of animal models, making it difficult to accurately predict personalized antigen presentation in humans [206]. Moreover, epigenetic agents such as HDAC inhibitors show robust activation of interferon signaling in models, yet their clinical efficacy may be attenuated by epigenetic heterogeneity and intrinsic tumor resistance in patients [186,187]. Therefore, future research should prioritize precision targeting strategies informed by multi-omics integration, alongside the optimization of drug delivery systems and the development of more physiologically relevant humanized models, to address translational bottlenecks and advance the clinical application of immune therapies for cold tumors.

### 4.4 Challenges and Future Directions

Immunotherapy for cold tumors is undergoing a pivotal transition from basic research to clinical translation. The integration of multi-omics technologies offers a transformative framework for elucidating the intricate mechanisms of immune escape; however, the precise modulation of the tumor immune microenvironment—from “cold” to “hot”—still faces numerous unresolved challenges. The pronounced spatial and temporal heterogeneity of cold tumors limits the effectiveness of traditional single-sample and single-cell sequencing approaches in capturing their dynamic immune landscapes. This necessitates the development of more advanced time-resolved multi-omics techniques and biomimetic tumor microenvironment models. A central challenge in cross-platform and cross-scale data integration lies in discrepancies in spatial resolution and cellular coverage, which directly impact our ability to accurately delineate immunosuppressive mechanisms. In response, cutting-edge computational frameworks, such as digital twin (DT) models for cancer patients, are leveraging high-performance computing and multimodal databases to shift the research paradigm from population-level inference to individualized simulation [207]. As a crucial enabler of precision medicine, the advancement of digital twin technology is being driven by artificial intelligence (AI) and machine learning algorithms, which hold promise for the personalized optimization of diagnostic and therapeutic strategies [208]. However, within the domain of immuno-oncology, the broader application of DT models is currently limited by challenges in the physiological fidelity of virtual patient simulations. To enhance predictive accuracy, there is a pressing need to integrate public resources—such as the Interactive Atlas for Immuno-Oncology Research—and adopt multi-omics and interdisciplinary approaches [209]. Looking ahead, the future of multi-omics integration should emphasize the optimization of clinical interpretation of biological variant data, support dynamic monitoring of laboratory indicators and tumor biomarkers, and facilitate the clinical translation of DT technologies [210]. In this context, precision oncology, empowered by AI, seeks to decode complex cancer signaling networks through the integration of whole-slide digital pathology and multi-omics datasets. Despite ongoing challenges in validation systems, regulatory approval, and standardization, this integrative framework is poised to significantly accelerate the development of biomarker-guided individualized therapies [211].

To achieve effective clinical translation for cold tumors, it is essential to adopt a dual-track strategy that integrates dynamic monitoring with precision intervention. Multi-omics-based molecular typing technologies are crucial for real-time tracking of treatment responses and regimen optimization, while smart therapeutic platforms must combine synthetic biology and nanotechnology to sense and respond to cues within the tumor microenvironment. Synthetic biology tools, such as chimeric antigen receptors (CARs), have demonstrated remarkable efficacy in hematologic malignancies; however, their application in solid tumors remains hindered by multiple immunosuppressive mechanisms and challenges in tumor infiltration and persistence [212]. Future advances will depend on the development of more predictive preclinical models, informed by fundamental immunological insights, to achieve a balance between therapeutic efficacy and safety [213]. Simultaneously, innovation in biomaterials should prioritize low-cost manufacturing, non-invasive monitoring, and *in vivo* controllability to accelerate the clinical implementation of synthetic immune interfaces [214]. Notably, although multi-omics analyses have successfully constructed melanoma prognostic models, the functional roles and dynamic regulation of key genes such as AGPAT2 within the immune microenvironment remain to be fully elucidated [215]. The identification of promising targets—such as small nuclear ribonucleoprotein polypeptide E—requires systematic validation using integrated approaches like CRISPR-based screening in combination with single-cell histological profiling [216]. Furthermore, while mitophagy-related prognostic models show strong predictive power, the cell-type-specific regulatory networks underlying these signatures still require high-resolution delineation through single-cell RNA sequencing [217].

Breakthroughs in spatial multi-omics have offered novel insights into intratumoral heterogeneity. For example, digital spatial analysis has demonstrated that proteomic signaling within the stromal region holds superior predictive value for response to bispecific antibody therapy [218]. Additionally, computed tomography-derived tumor habitat models have successfully predicted responses to neoadjuvant immunochemotherapy in patients with non-small cell lung cancer [219]. These findings underscore the critical importance of integrating imaging histology with multi-omics data. Ultimately, overcoming immune escape in cold tumors demands both technological innovation and conceptual advancement. By establishing a unified data framework supported by intelligent algorithms, we can drive transformative progress across the entire continuum—from mechanistic understanding to clinical intervention—and thereby usher precision immunotherapy into a new era of individualized treatment.

## 5 Conclusion

This review systematically delineates the immune evasion mechanisms underlying cold tumors and highlights multi-omics technologies as transformative tools for decoding tumor–immune interactions. By integrating genomics, transcriptomics, proteomics, metabolomics, and spatial omics, both intrinsic tumor resistance and extrinsic microenvironmental suppression are revealed as cooperative factors shaping immune escape. Key pathways such as WNT/β-catenin and TGF-β signaling consistently emerge as immunosuppressive axes, while metabolic and spatial reprogramming further reinforce immune exclusion. Multi-omics–based interventions—including immune checkpoint blockade combinations, metabolic targeting, and personalized neoantigen therapies—offer rational strategies to reprogram the tumor immune microenvironment. Nonetheless, challenges remain in cross-platform data integration, mechanistic causality, and clinical translation. Moving forward, the integration of dynamic multi-omics profiling with functionally validated models and intelligent therapeutic design is essential to overcome immune resistance and facilitate the transformation of cold tumors into immunologically active states.

## Data Availability

Not applicable.

## Highlights

- Multi-omics reveals immune evasion mechanisms in cold tumors
- Targeting WNT/β-catenin and TGF-β restores immune surveillance
- Combination therapies boost immune checkpoint inhibitor efficacy
- Personalized neoantigen vaccines and cell therapies advance treatment
- Clinical translation faces data heterogeneity and toxicity hurdles

## Abbreviations

AHR: Aryl hydrocarbon receptor
AIM2: Absent in Melanoma 2
Akt: Protein Kinase B
APC: Adenomatous Polyposis Coli protein
ATF3: Activating transcription factor 3
ATP: Adenosine Triphosphate
BC: Breast cancer
Bim: Bcl-2 interacting mediator of cell death
BIRC2: Baculoviral IAP Repeat-Containing Protein 2
Blimp1: B lymphocyte-induced maturation protein 1
BTLA: B and T Lymphocyte Attenuator
CAF: Cancer-associated fibroblasts
CAFs: Cancer-associated fibroblasts
CAR: Chimeric antigen receptor
CAR-T: Chimeric Antigen Receptor T-Cell
CBX2: Chromobox 2
CCAR1: Cell Division Cycle and Apoptosis Regulator 1
CCL: Chemokine (C-C Motif) Ligand
CCL4: Chemokine (C-C Motif) Ligand 4
ccRCC: Clear cell renal cell carcinoma
CD: Cluster of Differentiation
CD8^+^ T cells: Cytotoxic T lymphocytes
cDC1: Conventional Dendritic Cell 1
cDCs: Conventional dendritic cells
CH25H: Cholesterol 25-hydroxylase
CIBERSORT: Cell-type Identification By Estimating Relative Subsets Of RNA Transcripts
CITE: Cellular Indexing of Transcriptomes and Epitopes by Sequencing
CNVs: Copy number variations
COPD: Chronic obstructive pulmonary disease
CRC: Colorectal cancer
CRISPR: Clustered Regularly Interspaced Short Palindromic Repeats
CRISPRi: CRISPR interference
CSC: Cancer stem cell
CSF1R: Colony Stimulating Factor 1 Receptor
CTCs: Circulating tumor cells
CyTOF: Cytometry by Time-Of-Flight
cGAS-STING: Cyclic GMP-AMP Synthase-Stimulator of Interferon Genes
DAMPs: Damage-Associated Molecular Patterns
DC: Dendritic cell
DCs: Dendritic cells
DKK1: Dickkopf-1
EMT: Epithelial-mesenchymal transition
ERK1/2: Extracellular Signal-Regulated Kinase 1/2; ESCC, Esophageal squamous cell carcinoma
ETV4: Ets Variant 4
exoPD-L1: exosomal PD-L1
EZH2: Enhancer of Zeste Homolog 2
FTO: Fat mass and obesity-associated protein
FTSJ3: FtsJ RNA 2^′^-O-methyltransferase 3
GABA: γ-aminobutyric acid
GAD1: Glutamate decarboxylase 1
GBC: Gallbladder cancer
GBM: Glioblastoma
GC: Gastric Cancer
GLS: Glutaminase
GSK3β: Glycogen Synthase Kinase 3 Beta
HCC: Hepatocellular carcinoma
HDAC: Histone Deacetylase
HER2: The ErbB2 receptor tyrosine kinase
HGSOC: High-grade serous ovarian cancer
HIF-1α: Hypoxia-Inducible Factor 1 Alpha
HLA: Human Leukocyte Antigen
HLA-I: Human Leukocyte Antigen Class
IAV: Influenza A virus
I-BET151: Inhibitor of BET Protein
ICIs: Immune checkpoint inhibitors
ICB: Immune checkpoint blockade
ICC: Intrahepatic cholangiocarcinoma
IDO1: Indoleamine 2,3-dioxygenase 1
IFN: Interferon
IFN-γ: Interferon-γ
IEV: Immune evasion
IEVSig: Immune evasion signature
IL: Interleukin
IL-10R: Interleukin-10 receptor
IL-6R: Interleukin-6 receptor
ISG: Interferon-stimulated genes
ITF2357: Inhibitor of Histone Deacetylase
ITH: Intratumoral heterogeneity
LAG-3: Lymphocyte Activation Gene 3
LCR: Long control region
LEC: Lymphatic endothelial cell
LC-MV: Lymphocytic Choriomeningitis Virus
LGALS9: Galectin-9
LM: Liver metastasis
LOH: Loss of heterozygosity
LOY: Y chromosome loss
LRP1: Low-Density Lipoprotein Receptor-Related Protein 1
LSCC: Lung squamous cell carcinoma
LUAD: Lung adenocarcinoma
MC: Mucinous adenocarcinoma
MCF-7-CM: MCF-7 breast cancer cell secretome
MCP-counter: Microenvironment Cell Populations-counte
MDSCs: Myeloid-derived suppressor cells
METTL3: Methyltransferase Like 3
Mfge8: Milk Fat Globule-EGF Factor 8
MHC: Major histocompatibility complex
MHC-I: Major histocompatibility complex class I
MIF: Migration inhibitory factor
MISTRG6: Mouse model with human M-CSF, IL-3, GM-CSF, SIRPα, ThPO, and IL-6 genes
MMP9: Matrix Metallopeptidase 9
MM: Multiple myeloma
mregDC: Mature regulatory dendritic cells
NFAT: Nuclear factor of activated T cells
NFATC2: Nuclear Factor of Activated T-cells, Cytoplasmic 2
NF-κB: Nuclear Factor kappa-light-chain-enhancer of activated B cells
NHS-IL12: NHS76 Antibody-Human IL-12 Fusion Protein
NHA: Normal human astrocytes
NK: Natural killer cells
NKGS: NK gene signature
NKTCL: NK/T cell lymphoma
NOTCH: Notch Homolog
NR4A1: Nuclear Receptor Subfamily 4 Group A Member 1
NTRK1: Neurotrophic Receptor Tyrosine Kinase 1
NSCLC: Non-small cell lung cancer
NY-ESO-1: New York Esophageal Squamous Cell Carcinoma-1
OCCC: Ovarian clear cell carcinoma
ONECUT2: One Cut Homeobox 2
PABPC1L: Poly(A) Binding Protein Cytoplasmic 1 Like
PAMPs: Pathogen-Associated Molecular Patterns
PBAF: Polybromo-associated BRG1-associated factor complex
PDA: Pancreatic Ductal Adenocarcinoma
PD-1/PD-L1: Programmed Death-1/Programmed Death-Ligand 1
PD-L1: Programmed cell death ligand 1
PDAC: Pancreatic ductal adenocarcinoma
PDXs: Patient-derived xenografts
PI3K: Phosphoinositide 3-Kinase
PTPN1: Protein tyrosine phosphatase non-receptor type 1
RPS3: Ribosomal protein S3
R/R: Relapsed/refractory
RTKN2: Rhotekin 2
SASP: Senescence-associated secretory phenotype
scRNA-seq: Single-cell RNA sequencing
SCNA: Somatic copy number alterations
SCD1: Stearoyl-CoA Desaturase 1
sCNV: Somatic copy number variation
SMARCAL1: SWI/SNF Related, Matrix Associated, Actin Dependent Regulator of Chromatin, Subfamily a, Member 1
SOCS3: Suppressor of cytokine signaling 3
SOX2: SRY-Box transcription factor 2
STAT1: Signal transducer and activator of transcription 1
STING: Stimulator of interferon genes
TAM: Tumor-associated macrophage
TAMs: Tumor-associated macrophages
TBK1: TANK-binding kinase 1
TCGA: The Cancer Genome Atlas
Tcf1: T cell factor 1
TCR-T: T-cell receptor engineered T cells
TDO: Tryptophan 2,3-Dioxygenase
Tfh: T follicular helper
TFHL: T follicular helper lymphoma
TGF-β: Transforming Growth Factor-beta
TILs: Tumor-infiltrating lymphocytes
TIME: Tumor immune microenvironment
TLS: tertiary lymphoid structures
TLR: Toll-like receptor
TMB: Tumor mutational burden
TME: Tumor microenvironment
Tregs: Regulatory T cells
VEGF: Vascular Endothelial Growth Factor
VEGF-A: Vascular Endothelial Growth Factor-A
WNT: Wingless
WNT2: Wingless-Type MMTV Integration Site Family Member 2
YAP1: Yes-associated protein 1
ZBTB46: Zinc Finger and BTB Domain-Containing Protein 46
